# Scalable Paper Supercapacitors for Printed Wearable Electronics

**DOI:** 10.1021/acsami.2c15514

**Published:** 2022-12-12

**Authors:** Mehmet Girayhan Say, Calvin J. Brett, Jesper Edberg, Stephan V. Roth, L. Daniel Söderberg, Isak Engquist, Magnus Berggren

**Affiliations:** † Laboratory of Organic Electronics, Department of Science and Technology, Linköping University, SE-601 74Norrköping, Sweden; ‡ Wallenberg Wood Science Center, KTH Royal Institute of Technology, Teknikringen 56-58, 100 44Stockholm, Sweden; § Department of Engineering Mechanics, KTH Royal Institute of Technology, Osquars Backe 18, 100 44Stockholm, Sweden; ∥ Deutsches Elektronen-Synchrotron (DESY), Notkestrasse 85, 22607Hamburg, Germany; ⊥ RISE Research Institutes of Sweden, Bio- and Organic Electronics, Bredgatan 35, SE-602 21Norrköping, Sweden; # Fibre and Polymer Technology, KTH Royal Institute of Technology, Teknikringen 56-58, 100 44Stockholm, Sweden; ∇ Wallenberg Wood Science Center, ITN, Linköping University, SE-601 74Norrköping, Sweden

**Keywords:** nanocellulose, PEDOT:PSS, spray coating, GIWAXS, GISAXS, supercapacitors, wearable electronics

## Abstract

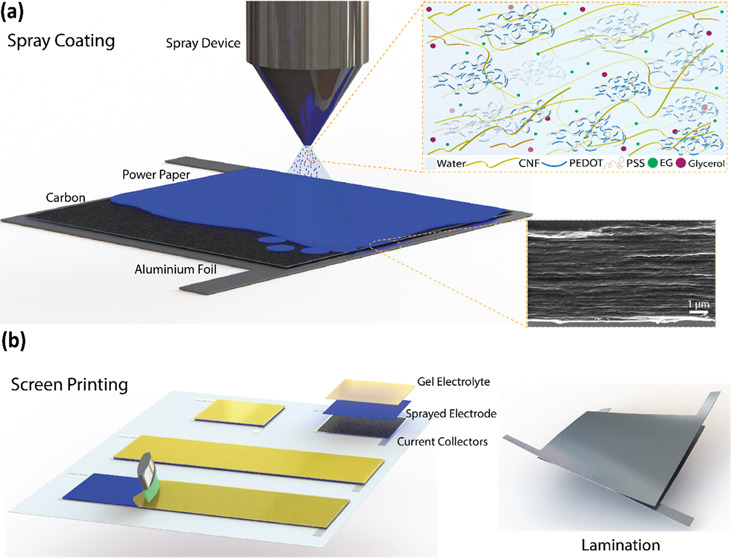

Printed paper-based electronics offers solutions to rising energy concerns by supplying flexible, environmentally friendly, low-cost infrastructure for portable and wearable electronics. Herein, we demonstrate a scalable spray-coating approach to fabricate tailored paper poly(3,4-ethylenedioxythiophene):poly(styrene sulfonate) (PEDOT:PSS)/cellulose nanofibril (CNF) electrodes for all-printed supercapacitors. Layer-by-layer spray deposition was used to achieve high-quality electrodes with optimized electrode thickness. The morphology of these electrodes was analyzed using advanced X-ray scattering methods, revealing that spray-coated electrodes have smaller agglomerations, resulting in a homogeneous film, ultimately suggesting a better electrode manufacturing method than drop-casting. The printed paper-based supercapacitors exhibit an areal capacitance of 9.1 mF/cm^2^, which provides enough energy to power electrochromic indicators. The measured equivalent series resistance (ESR) is as low as 0.3 Ω, due to improved contact and homogeneous electrodes. In addition, a demonstrator in the form of a self-powered wearable wristband is shown, where a large-area (90 cm^2^) supercapacitor is integrated with a flexible solar cell and charged by ambient indoor light. This demonstration shows the tremendous potential for sequential coating/printing methods in the scaling up of printed wearables and self-sustaining systems.

## 1 Introduction

Wearable electronics and the Internet of things (IoT) hold great potential for smart systems, where active and passive components are connected into self-powered systems that are flexible and thin (<1 mm).^1,2^ The need for self-powered systems is rapidly expanding in various applications from continuous health monitoring, energy management, and smart homes to prosthetics.^3,4^ All of these systems require intermediate energy storage modules, in the form of flexible or stretchable rechargeable batteries or supercapacitors, efficiently integrated with energy harvesting technologies (i.e., photovoltaics, piezoelectric and triboelectric nanogenerators) to achieve continuous operation.^5−7^ Since, supercapacitors offer excellent cycle performance, high peak power, safe operation, and exhibit high tolerance toward fluctuating charging currents, they are being regarded as excellent candidates to sustain the long-term operation of self-powered flexible systems.^8,9^ Remaining challenges are the lack of alternative fabrication methods for large-scale production, high-quality electrode manufacturing, and the design of safe electrochemical devices from environmentally friendly polymeric materials. In a recent publication, we have identified combined cellulose nanofibril (CNF)/(poly(3,4-ethylenedioxythiophene)):poly(styrene sulfonate) (PEDOT:PSS) electrodes, manufactured by spray coating, as a promising concept for flexible paper-based supercapacitors with good performance.^10^ Here, we will expand this concept to larger areas while maintaining high electrode quality and performance, as proven by in situ morphological characterization during deposition.

Paper-based energy storage devices are becoming a key technology, using paper either as a flexible and low-cost substrate or by incorporating cellulose fibrils as a structural element into the device architecture (electrode, electrolyte, separator membranes) to provide mechanical strength and functionality.^11,12^ The combination of electroactive materials with paper-based polymeric networks (cellulose fibrils, pulp, and paper substrates) generates ample opportunities to achieve mechanically robust, lightweight, and low-cost electrodes for energy systems.^13,14^ The materials can be formulated into inks for printing, enabling the manufacture of each layer (electrode, electrolyte) on top of one another and thus to print the device in a thin and lightweight form with high yield.^14^

Solution-processed printing techniques are of the greatest importance in scaling up the fabrication of paper-based and organic electronic devices, facilitating to shift from prototypes to low-cost consumer electronics applications.^12^ Lab-scale film fabrication techniques such as drop-casting, spin coating, and dip coating are the traditional methods to study the proof-of-concept devices but lack the ability to provide either conformal coating or large-area coverage. Screen printing provides excellent volume manufacturing capability, has been shown with both CNF-based and cellulose pulp-based inks, and is highly used to fabricate thick supercapacitor electrodes.^15−17^ However, it lacks the ability to cover nonflat surfaces in a conformal way and may, depending on the application, result in unnecessarily high electrical interface resistance and limited flexibility. One printing/coating technique that could potentially address these issues is spray coating, which has shown to be convenient in producing scalable thin films of nanomaterials or conformal ultrathin coatings for the healthcare and food industry, as well as conductive composites.^18,19^ For energy storage technology, airbrush-assisted spray coating enables to deposit several hundreds of nanometers of electroactive films, using carbon-based, inorganic materials and conductive polymers.^20−22^ The handheld airbrush spray coating allows for simple manufacturing; however, the droplet volume is high, leading to bigger agglomerations. Further, it allows less control of the thickness and drying patterns, and lastly, it suffers from fast clogging even for high-pressure gas-driven systems.^23^ To overcome these drawbacks, industrial air atomizing and ultrasonic spray technology are introduced, where ultrafine droplets with narrow size distribution and limited material loss can be created even with low gas pressure, achieving smooth films with large-area coverage and roll-to-roll compatibility due to the automatized systems.^23,24^ Using CNF as a nanoscale binder provides a nanoporous polymeric scaffold, high surface area, and porosity, leading to high mass loadings throughout the film, and therefore opens a pathway to spray coat nanomaterials with a high control capability on the electrode thickness.^10,25,26^

A variety of different conductive polymers with pseudocapacitive properties, such as polyaniline (PANI), polypyrrole (PPy), and PEDOT, have been incorporated into flexible scaffolds (paper, fibrils, etc.) to form electrodes for flexible batteries and supercapacitors.^12,27,28^ In particular, PEDOT-based materials have attracted interest in many diverse fields because of their high capacitance (from 0.99 μF/cm^2^ to 2.24 F/cm^2^), high conductivity (∼1000 S/cm), and low impedance; strong examples are found in, e.g., biomedical and energy storage applications.^29−31^ In energy storage applications, PEDOT can be used as a capacitive coating material for Li-battery cathodes and as high-capacity electrode material for supercapacitors, where the material can be produced with both physical deposition techniques and polymerization methods such as oxidative chemical vapor deposition (oCVD) and electropolymerization.^31,32^ PEDOT can also be polymerized into the paper-based systems,^33,34^ but since such paper electrodes are integrated into supercapacitor structures with lamination, the devices will exhibit high equivalent series resistance (ESR), limited bending stability, and bending radius. Further, the fabrication methods limit the produced area and the mechanical robustness.^30,35,36^ In response to these drawbacks, water-dispersible PEDOT:PSS, which is a commercially available polymeric ion–electron conductor with excellent rheological properties, is an attractive alternative. PEDOT:PSS has been used to scale up batch processes with paper-based materials to achieve the goals of fully printed paper energy storage systems, demonstrated in use cases and flexible electronics applications.^10,26,37^ Following these efforts, considerable attention has been given to understanding and optimizing the interface and bulk morphology of PEDOT electrodes. Recently, the morphological change during drying and film formation and shift in the π–π stacking distance of PEDOT, as well as changes in the device performance metrics, i.e., conductivity and mobility, have been investigated via the effect of additives (cosolvents, surfactants) and method of printing.^38,39^ In addition, paper-based PEDOT nanocomposite electrodes have been addressed to characterize the effects of environmental changes on the crystallization of PEDOT during film formation and self-assembly around a one-dimensional (1D) nanoscale biotemplate cellulose matrix.^25,40^ Powerful investigation tools here are the synchrotron-based characterization techniques, which have been used to reveal the nanoscale morphology at the interfaces and the active layers of energy technologies.^41,42^ Changes in crystallinity have been investigated for polymeric systems manufactured with different printing techniques, with the aim to achieve optimum performance by understanding the morphology as well as kinetics, especially in conductive polymers.^38,41^ Considerable attention has been given to PEDOT systems, which have been investigated intensively to understand structural kinetics better during electrochemical doping and dedoping.^43^ However, there is still a need to investigate large-scale homogeneity and morphological changes when a roll-to-roll (R2R) compatible printing/coating technology is introduced.^44^

In this study, we introduce an optimized paper electrode (PEDOT:PSS/CNF) fabrication process using an air-atomized spray-coating technique, to be implemented into flexible, large-area paper supercapacitors. Electrodes with superior control on the thickness and potential for scalability are demonstrated, covering both screen printing and spray coating-based fabrication limits to achieve electrodes ranging from 1.7 to 30 μm. We combine the industrial scale, fast spray deposition process with grazing incidence wide/small-angle X-ray scattering methods (GIWAXS/GISAXS), to investigate large-scale homogeneity assisted with advanced surface mapping. In particular, the morphology, microstructure, and crystallinity of spray-coated paper electrodes are analyzed and compared with the traditional electrode-making process, drop-cast films. The fabrication methods are compared, and differences in aggregation mechanisms are explained with different drying kinetics. Flexible, all-solid-state paper electrodes are assembled as supercapacitors using sequential coating/printing and electrical and mechanical characterization is performed. We also show how these supercapacitors can be integrated and operated with electrochromic displays and flexible solar cells, to demonstrate the potential for applications such as self-powered, wearable systems.

## 2 Experimental Section

### 2.1 Reagent and Materials

PEDOT:PSS (PH1000, 1.3 wt %) was purchased from Heraeus Clevios GmbH. Ethylene glycol, glycerol, hydroxyethyl cellulose (HEC, *M*_w_ = 250 000), and 1-ethyl-3-methylimidazolium ethyl sulfate (EMIM-ES, >95%) were purchased from Sigma-Aldrich; 0.52 wt % CNF dispersed in water (degree of polymerization is around 500 and degree of crystallinity is around 40%) was purchased from RISE Bioeconomy. Al/PET current collectors were provided by DPP AB, and carbon conductive paste (Microcircuit Materials 7102) was purchased from Dupont. Distilled water (DI) was used as the solvent in all ink preparations.

### 2.2 Ink Preparation

Paper electrode ink was prepared by mixing CNF, PEDOT:PSS, and the additives at room temperature. First, 0.52 wt % CNF solution in water was diluted down to 0.1 wt %. PEDOT:PSS was mixed with EG (5 wt %); 23 g of 0.1 wt % CNF dispersion was mixed with 5 g of PEDOT:PSS solution and 0.2 g of glycerol was slowly added to the mixture. The ink was homogenized with an Ultra-Turrax shear mixer for 5 min and then degassed for 1 h.

For the printed gel electrolyte ink, 3.5 g of methylimidazolium:ethyl sulfate (EMIM:ES) was mixed with 11.5 g of DI water vigorously mixed, and then 1 g of HEC was added to solution. The solution was mixed at 90 °C for 1 h to become a clear gel.

### 2.3 Spray Coating of the Paper Electrode

The spray deposition experiments were performed using an industrial automatized spray setup. The spray deposition nozzle was a Compact JAU D555000 (Spray Systems Inc.), which was controlled with magnetic valves to open and close individual spray cycles. The sampling liquid was attached to a 12 mL siphon container at the spray nozzle and the driving gas was nitrogen at 1 bar with a flow of 30 L/min. The distance between the nozzle and sample surface was set to be 200 mm. Beneath the foil substrate, we used a vacuum hot plate (EMS 1000 series, Electronic Microsystems) where we annealed the substrate during the spray deposition to 95 °C. To cover the large surface area on the sample, we used a motorized linear stage (LTS300/M, Thorlabs Inc.) to move the spray nozzle across the sample surface. We moved on the 220 mm range with a speed of 20 mm/s and an acceleration of 20 mm/s^2^ back and forth. The spray deposition was done during the movement crossing the sample surface with a pulsed operation mode. The ink was sprayed for 50 ms and for 950 ms the nozzle was closed till the next spray pulse opened. We repeated the opening and closing routine up to 22 times per sample, which resulted in a single-layer deposition. For multilayer films, this step was automatized to deposit up to 300 layers.

### 2.4 Drop-Cast Electrodes

The same ink used in spray coating is drop-cast to fabricate paper electrodes. For X-ray characterization of samples, the ink was drop-cast on the heated substrate by keeping the same experimental parameters (in the chamber, temperature and humidity) as in the spray-coating setup. For device comparison, the ink was poured into a Petri dish (5 cm diameter) and baked in an oven overnight at 70 °C, and then annealed at 95 °C for an hour. The film was peeled off from the substrate, cut into appropriate dimensions, and placed on the Al/C current collectors. Then, the gel electrolyte was screen printed on the paper electrodes, and identical parts are laminated to have a full-cell configuration. For all samples, the thickness of the deposited films was measured to maintain a control experiment.

### 2.5 Printed Device Assembly

Several printing/coating procedures follow each technique to produce solid-state, flexible supercapacitors. We used commercially available thin aluminum sheets laminated on flexible plastic substrates as current collectors. A layer of screen-printed carbon was added on top of the aluminum to protect the metal from corrosion as well as to promote adhesion between the current collector and the electrode layer. First, carbon paste was printed on the patterned Al/Pet foil and dried at 60 °C for 1 h, and then the CNF/PEDOT:PSS ink was deposited using an automated spray-coating system on the substrates that were placed on a hot plate at 95 °C. After achieving the desired electrode thickness, the electrolyte slurry was deposited by screen printing. After being dried overnight, the half-cells were laminated to become a full-cell supercapacitor and pressed with a pressure of 0.2 MPa for better adhesion.

### 2.6 Characterization Methods

Scanning electron microscopy (SEM) was conducted by Sigma 500 Gemini (Zeiss). Atomic force microscopy (AFM) measurements were conducted on a MultiMode MMAFM-2 (Bruker Corporation) using a silicon nitride cantilever designated for soft materials (ScanAsyst-AIR, resonance frequency: 70 kHz, spring constant: 0.4 N/m, nominal tip radius: 2 nm, Bruker Corporation, USA). The ScanAsyst tapping mode was used to topographically map (1 × 1) μm^2^ sample surfaces. The rheological property of the electrode ink was measured by an Anton Paar MCR 102.

Optical microscopy images were taken using a Zeiss microscope. Conductivity measurements were made using an Ossila Four Point Probe System. Thickness values were measured with a Dektak and confirmed with an optical profilometer Plux NeoX (Sensofar).

GISAXS and GIWAXS are powerful techniques used to reveal information from nanocomposites, specifically molecular ordering and particle structure.^45−47^ These techniques allow us to study the inner morphology of the deposited film, where orientation and particle formation changes can be observed for the films that are manufactured by different fabrication methods. The GISAXS and GIWAXS experiments were conducted at the P03/MiNaXs beamline at PETRA III (Hamburg, Germany). The sample-to-detector distance SDD was SDD_GISAXS_ = (2644 ± 1) mm and SDD_GIWAXS_ = (128.5 ± 0.2) mm for GISAXS and GIWAXS, respectively. The beam size was (32 × 15) μm^2^ and the X-ray energy was 12.58 keV calibrated using collagen and lanthanum hexaboride. The scattered intensity was recorded using a Pilatus 1 M ([172 × 172] μm^2^, Dectris Ltd., Switzerland) in GISAXS and a Pilatus 300 k ([172 × 172] μm^2^) in GIWAXS geometry. The samples were either spray-deposited or drop-cast. The spray deposition was done by spraying 60 times for 0.1 s and intermittent drying. The drop-casting was done using a pipette and 50 μL of the same ink as in spray deposition. For both cases, the silicon wafers were piranha cleaned, as reported elsewhere.^47^ The samples were measured for 100 ms at 10 positions to check for homogeneity and to distribute the X-ray dose. All scattering patterns were summed up for higher statistics. The GISAXS patterns were analyzed using DPDAK v.1.4.1 and the GIWAXS patterns using GIXSGUI v.1.7.1.^48,49^ The one-dimensional integration along the Yoneda region of the ink was fitted using a Guinier–Porod model to extract lateral length scales as well as their corresponding morphologies.^50^

### 2.7 Electrochemical Analysis

All of the electrochemical characterizations were performed using a Biologic 200 potentiostat/galvanostat. Self-discharge performance was recorded using a Keithley 2400 source meter with custom-made Labview software. The scan rate for cyclic voltammetry was applied from 5 mV/s to 500 mV/s.

The capacitance and equivalent series resistances (ESR) were extracted from the GCD measurements using eqs 1 and 2, respectively
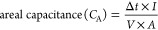
1
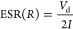
2where Δ*t*, *I*, *V*_d_, and *V* are the discharge time, discharge current, voltage drop at the beginning of the discharging (iR drop), and difference between operation voltages, respectively.

The power and energy densities were calculated according to the formulas in eqs 3 and 4, respectively.
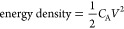
3
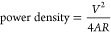
4where *A* is the electrode area (cm^2^), *C*_A_ is the areal capacitance, *V* is the operating voltage, and *R* is the ESR.

The overall efficiency of the wearable photoelectrochemical energy conversion was calculated by eq 5
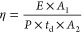
5where *E* is the energy density of the large-area supercapacitor calculated from the discharge curve, *A*_1_ is the area of the supercapacitor, *P* is the light energy density (∼0.1 mW/cm^2^), *t* is the photocharging time, and *A*_2_ is the area of the wearable solar cell.

## 3 Results and Discussion

**Figure 1:**
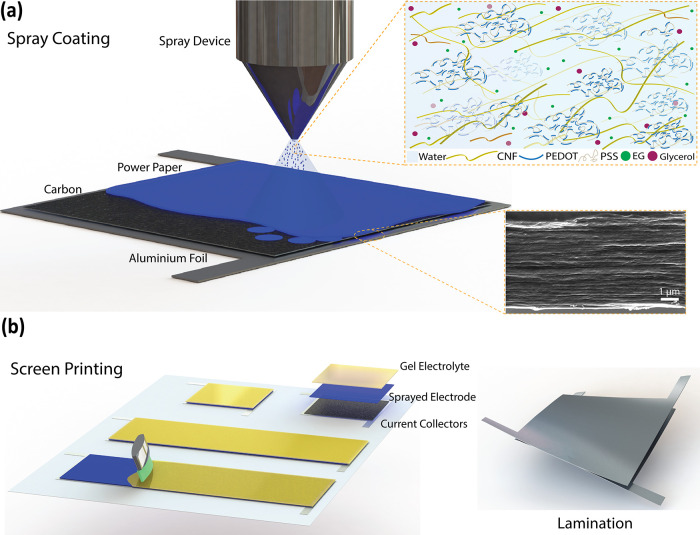
Schematic illustration of a mixed ion–electron conductor paper electrode and supercapacitor fabrication route. (a) Spray coating of the electrode on large surfaces, inset: cross-sectional SEM image of the spray-coated paper electrode and (b) screen printing of the gel electrolyte, and finally, lamination of printed layers to form a flexible paper-based supercapacitor.

The fabrication of a flexible and paper-based supercapacitor needs multiple production steps due to the designed functionalities of each layer. These functionalities stem from different material compositions for the different parts of the supercapacitors, e.g., current collectors, high surface area electrodes, and polymer electrolytes. Here, we show a route for the fabrication of industrially scalable production steps to produce printable, large-area supercapacitors. A combination of spray coating and other printing methods demonstrate up-scalable, large-area supercapacitors. Figure 1 illustrates the spray-coating procedure of the paper electrodes and sequential printing methods to fabricate flexible supercapacitor devices on current collectors; more technical details are provided in Section 2. Briefly, flexible current collectors (PET/Al/carbon) are defined as a 4.5 cm by 4.5 cm test cell configuration or 10 to 18 cm, as a large-area wearable device. Then, the paper electrode ink is spray-coated with an industrial air atomizing system. The typical ink for a spray-coated paper electrode consists of the conductive polymer PEDOT:PSS, cellulose nanofibrils (CNF), and certain additives used to enhance processing conditions and improve electrical performance. PEDOT:PSS is a low-cost, high-volumetric capacitance, and water-dispersible organic conductive polymer, which is utilized as an electroactive material. PEDOT:PSS is composited with cellulose nanofibrils (5–15 nm in diameter, 1–5 μm long), which provide a mechanically strong, nanoporous network to fabricate thick electrodes to realize paper electrodes. Organic additives such as ethylene glycol and glycerol (plasticizer) are introduced into the ink formulation to obtain conductive, soft polymeric electrodes and further provide flexibility.^51^ The prepared ink exhibits a typical shear thinning with a viscosity of ∼0.1 Pa.s at a shear rate of 1 s^–1^, which is in the range of spray-coating technology (Figure S1a, for contact angle analysis, see Figure S1b). The electrode layer is deposited using a spray-coating technique in a layer-by-layer (LbL) fashion to tune the thickness and control the active material mass. With this, the thickness was controlled by increasing the number of spray cycles^45^ (Figure 1a inset, cross-sectional SEM image, scale bar 1 μm). Then, a gel electrolyte composed of ionic liquid 1-ethyl-3-methylimidazolium ethyl sulfate (EMIM-ES) and hydroxyethyl cellulose (HEC) was screen-printed on the spray-coated paper electrodes as both the electrolyte and separator. Finally, the identical half-cells were laminated to obtain a flexible supercapacitor structure (Figure 1b).

### 3.1 Probing Morphology of the Electrode with Grazing Incidence X-ray Techniques

**Figure 2:**
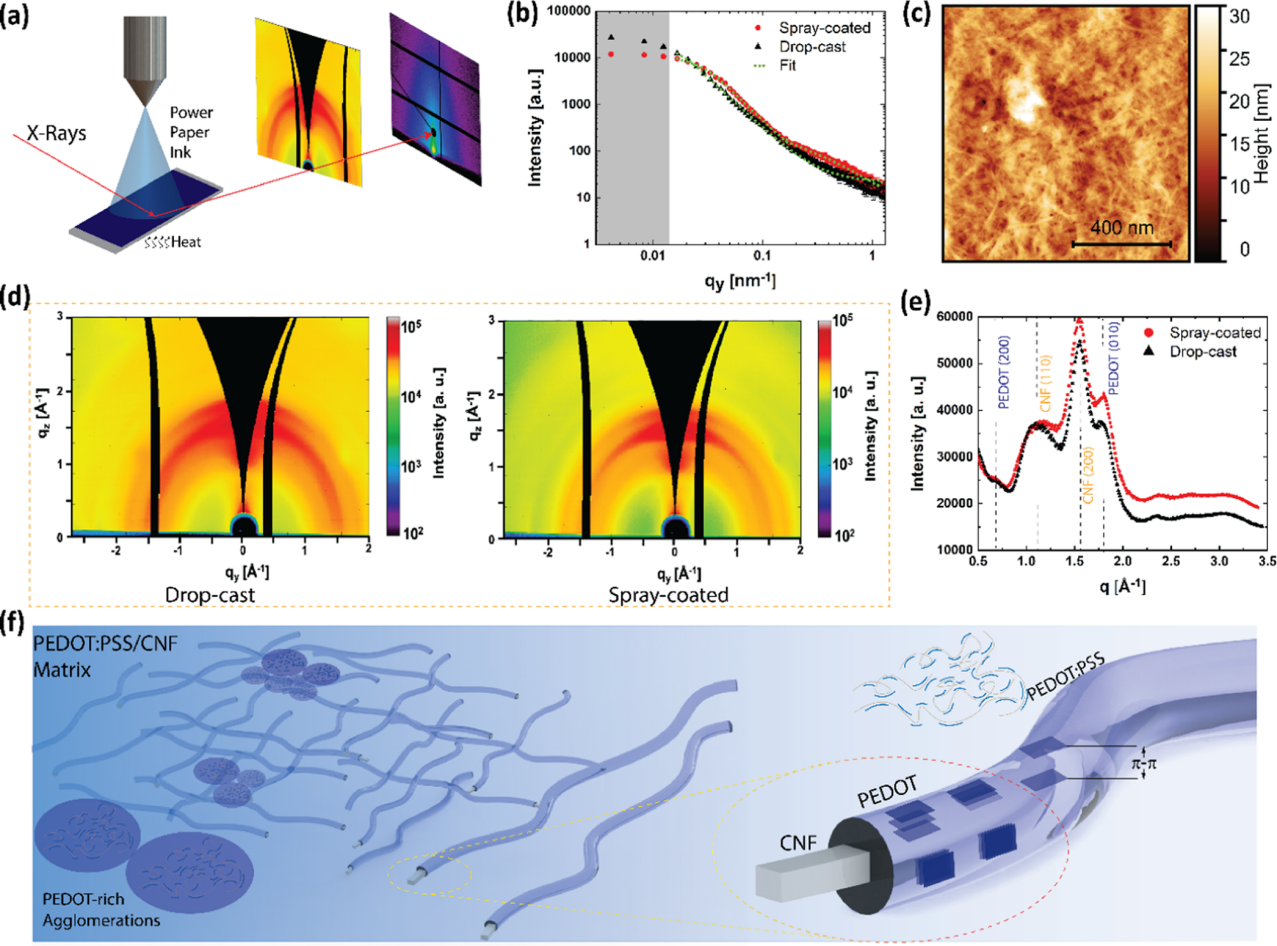
Structure and inner morphology of the electrode. (a) Schematics of grazing incidence small and wide-angle X-ray measurements conducted on the spray-coated paper electrode. (b) One-dimensional integrated GISAXS intensity at the Yoneda region of PEDOT:PSS together with a Guinier–Porod fit in two distinct regions to deduce the size features within a thin film. (c) AFM topography image of the spray-coated paper electrode. (d) Two-dimensional GIWAXS pattern of the spray-coated (left) and drop-cast (right) electrodes. (e) One-dimensional GIWAXS profile with the peaks assigned to PEDOT (200), CNF (110), CNF (200), and π-stacking of the PEDOT in the bio-nanocomposite. (f) Schematic representation of the spray-coated paper electrode.

The nanoscale morphology of the paper electrodes is investigated using grazing incidence X-ray techniques (Figure 2). A schematic of the spray-coating setup used in the synchrotron experiments is shown in Figure 2a, where a roll-to-roll spray-coating system deposits the ink on a current collector film. The composite electrodes were characterized using GISAXS and GIWAXS immediately after film formation, where the coating setup allows measuring the film at different stages. Figure 2b shows the one-dimensional intensity plotted along *q*_y_ for the GISAXS measurements for paper electrodes. Both spray-coated and drop-casted samples were analyzed using the same Guinier–Porod model to achieve optimum fitting.^52^ It should be noted that the Porod exponent *s* ≈ 2 hints at Gaussian polymer chains. *s* depends on the geometry and yields the form factor of the polymer: For 3D objects, i.e., spheres, *s* = 0, for rods *s* = 1, and in our case, it is a coiled structure, *s* = 2.^50^ The one-dimensional GISAXS data show clear differences at around 0.04 and 0.3 nm^–1^, comparing spray deposition and drop-casting, respectively (Table in S1). For the drop-cast film, a single feature size was observed: *R*_sph_ = 77.3 ± 0.1 nm. For the spray-deposited film, two distinct feature sizes were found: *R*_sph_,_sp._ = 47.9 ± 0.1 nm and *R*_cyc_ = 1.6 ± 0.1 nm. In both samples, we find spherical features *R*_sph_,_sp._ = (47.9 ± 0.1) nm and *R*_sph_,_dr._ = (77.3 ± 0.1) nm for spray-deposited and drop-cast, respectively.^45^*R*_sph_,_sp._ = 47.9 ± 0.1 can be attributed to PEDOT-rich agglomerations (clusters), which are in accordance with earlier studies, where agglomerations were explained in colloidal gel formation.^53,54^ The topographic image of spray-coated CNF/PEDOT:PSS demonstrates randomly distributed cellulose nanofibrils (surface roughness root mean square (RMS)) σ_rms_ = [13.5 ± 0.2] nm and excess PEDOT:PSS (agglomerations), which are self-organized around the carboxymethylated CNF matrix (Figure 2c). The Guinier–Porod fitting revealed that we gain smaller, more homogeneous spherical structures by spray deposition of around 48 nm, pointing to a more homogeneous distribution of PEDOT:PSS on the nanofibril network. For the spray-deposited sample that has the same thickness as the spray-coated electrode, we find a second structure, which has a cylindrical structure with a cross-sectional radius of *R*_cyl., sp._ = [1.6 ± 0.3] nm, which fits well with the characteristic length scale of the size of individual nanocellulose fibers. In addition, we resolve in the scattering experiments’ individual CNF fibers in spray deposition, and in the case of drop-casting, we find only a fully equilibrated larger structure incorporating the CNFs, meaning entangled CNF networks move away from the surface (see, AFM image in SI Figure S2, RMS roughness σ_rms_ = [16.5 ± 0.3] nm). On the contrary, spray deposition leads to nonequilibrium drying and immediate adhesion/deposition of particles and polymeric structures, partly preventing the self-assembly process and resulting in a confined morphology (reduced coffee ring effect, high uniformity, small agglomerations). Although the same CNFs are used in the drop-cast films, one can conclude that the self-assembly results in a more homogeneous film formation in the case of spray coating, see Table S1. The homogeneity stems from short drying processes after the removal of the solvent and smaller agglomerations since spray coating allows us to deposit in an LbL fashion. The two-dimensional (2D) GIWAXS patterns of both spray-coated and drop-cast films are shown in Figure 2d, where the increased intensity corroborates our findings in the favor of spray-coated electrodes. With this method, intermolecular stacking can be determined, and the scattering peaks of spray-coated and drop-cast samples exhibit crystalline and π–π packing peaks attributed to CNF ([110], [200]) and PEDOT ([200], [010]) (Figure 2e). The position of the PEDOT (010) π–π stacking is clearly observable at 1.8 Å^–1^ (real-space distance of *d* = 3.5 Å) for both techniques, which were reported in early studies for ethylene glycol-doped PEDOT:PSS.^38,54^ The spray-coated film exhibits better order (visible peaks for PEDOT), attributed to different drying kinetics. LbL deposition of spray coating of paper ink results in more uniform film coating, and low roughness (negligible coffee ring effect) enables homogeneous distribution of PEDOT-rich sites to maximize strong signals for PEDOT. The peaks are more pronounced for the spray-coated paper electrodes and have a lower angular extension. Based on the results and analysis, a schematic image can be proposed to picture the spray-coated paper electrode from the microscopic to the nanometer scale, see Figure 2f. From the left macroscopic side of the nanocomposite network, when it scales down the nm level, PEDOT-rich agglomerations are prominent in the CNF matrix, where on closer look, a single cellulose fibril is covered by the PEDOT:PSS coating. On the right-hand side, π–π stacking of PEDOT can be extracted and represented as crystallites for spray-coated nanopaper electrodes. From these findings, we can conclude that spray coating leads to smaller homogeneous structures and more prominent crystallites, leading to higher conductivity of the electrode. As a consequence, improved electrochemical performance is observed for the supercapacitor devices, which are fabricated both with spray coating and drop-casting. The details of the device comparison are explained in the electrochemical performance section (see Section 3.2.2).

### 3.2 Electrochemical Performance of the Paper Supercapacitors

#### 3.2.1 Effect of Electrode Thickness

**Figure 3:**
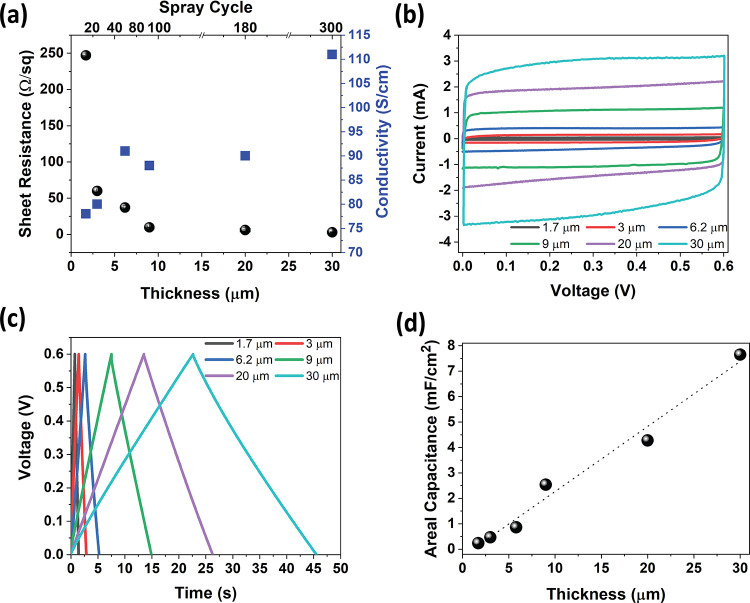
Electrical and electrochemical performance of the paper electrode. (a) Electrical characterization of the electrode. Sheet resistance vs conductivity of the paper electrodes with different thickness values. (b, c) CV at a scan rate of 20 mV/s and GCD at 0.2 mA/cm^2^ curves of supercapacitors with different electrode thicknesses, respectively. (d) Calculated areal capacitance at different electrode thicknesses.

To manufacture energy storage device electrode layers, the thickness of the electrode (amount of active material) can be tuned by the drop-casting volume or printing cycles of the processes. A summary of the electrical properties of the paper electrode and capacitance characterization of supercapacitor devices is given in Figure 3. Figure 3a shows conductivity and sheet resistance of the electrodes as a function of the number of spray cycles. The spray-coated paper electrodes maintain conductivity of more than 80 S/cm. The paper electrode conductivity is enhanced by doping the PEDOT:PSS with ethylene glycol, which results in low resistance. The sheet resistance varies with thickness, which leads to a slight increase in electrode conductivity. Sheet resistance saturates at the thicker films exhibiting 3.0 Ω/sq and a conductivity of 112 S/cm for the 30 μm film is achieved. A comparison table for other flexible electrodes is given in Table S2. To evaluate the electrochemical performance of printed devices with different electrode thicknesses, we fabricated identical, symmetrical supercapacitor test cells as demonstrated in Figure 1 and characterized them in a two-electrode configuration. Figure 3b displays the cyclic voltammogram (CV) of supercapacitors at 20 mV/s scan rates that have different paper electrode thicknesses. The rectangular shape and the symmetrical response of the CV curves imply an ideal capacitive behavior of the paper electrodes for all thickness values. Typical CVs are recorded, where PEDOT:PSS shows dominant capacitance contribution from volumetric double layers.^55^ To calculate the capacitance and evaluate the IR drop (voltage drop at the onset of discharge), galvanostatic charge–discharge (GCD) tests were performed at a moderate current level of 0.2 mA/cm^2^. Identical charge–discharge curves are recorded for devices with different thicknesses indicating balanced charge storage with a minimum 98.2% Coulombic efficiency, as shown in Figure 3c (Figures S4–S8). The curves are symmetrical for all thicknesses, representing the EDL-based fast charging–discharging with a quite low IR (ESR = IR_drop_/2*I*, eq 1) drop.^56^ Due to the superior contact between the current collectors and sprayed paper electrode, very low ESR values (6 to 12 Ω cm^2^) are calculated for all thickness values (Table S3). When it is compared, our printed devices show one order magnitude less ESR for the devices with different electrode thicknesses, with respect to the family of other electroactive materials. For instance, typical values are 11.52 Ω cm^2^ for graphene^21^ and 58.5 Ω cm^2^ for CNT^57^ layers obtained by spray coating and 9.0 Ω cm^2^ Mxene^58^ for supercapacitors, all designed as pouch cell configurations. Capacitance values for devices are calculated from galvanostatic charge–discharge (GCD) curves at a current density of 0.2 mA/cm^2^. It can be observed that the areal capacitance gradually increases as the thickness increases, indicating more active mass loading in the electrode with higher spray cycles. Figure 3d represents the linear trend of tuning the device’s capacitance while increasing the thickness, starting from 1.7 to 30 μm with an areal capacitance from 0.24 to 7.65 mF/cm^2^ at the 0.2 mA/cm^2^ GCD test, respectively. These values (7.65 mF/cm^2^, ∼26 F/g) are better or comparable to the previously published PEDOT-based supercapacitors^59−61^ (see Tables S4 and S5).

**Figure 4:**
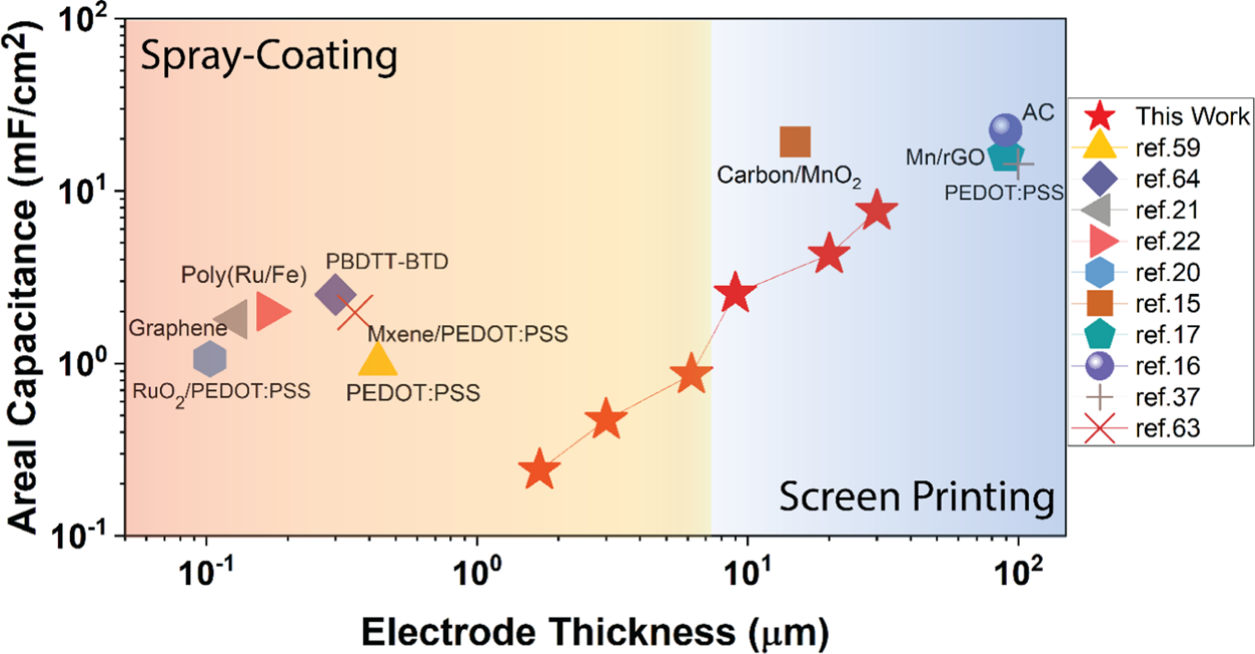
Comparison of screen-printing and spray-coating techniques, thickness vs areal capacitance.^63,64^

Figure 4 shows a comparison of the previous studies and this work by comparing spray coating and screen printing in terms of areal capacitance vs electrode thickness. One of the biggest challenges in the field of printed electronics is controlling the thickness of the active layer, where screen and stencil printing can cover ten to several hundreds of micrometers.^62^ Our method gives control on the capacitance by thickness adjustment, which can cover both spray-coating and screen-printing limits, and increasing the electrode thickness can be achieved with spray coating. Precision control on the thickness and area with printing-based manufacturing methods can respond to energy needs from low-power systems to complex high energy-requiring applications along with low ESR and, therefore, high-power peaks.

**Figure 5:**
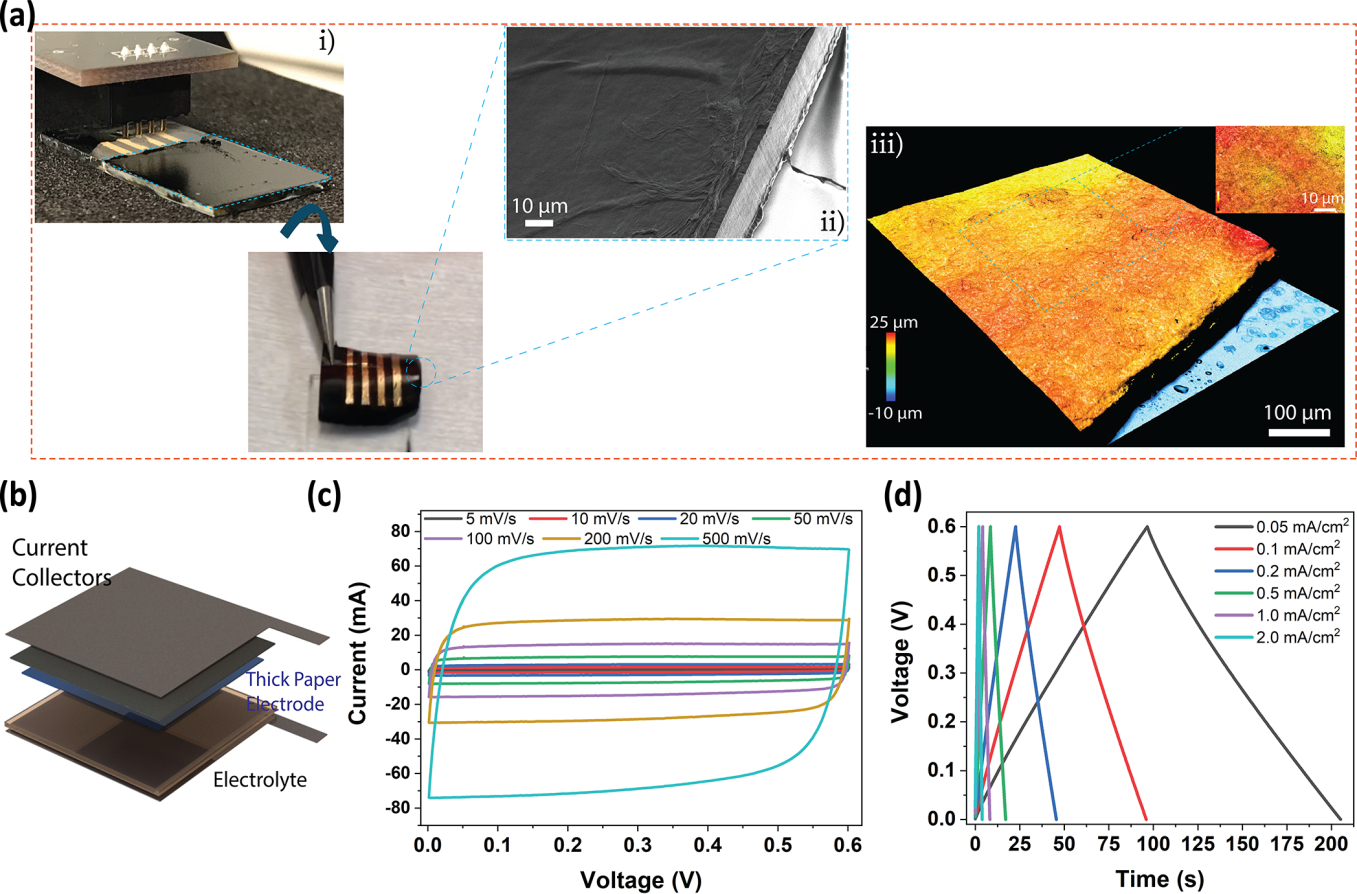
Electrode (30 μm thick) manufacturing and device performance. (a) Optical (i), SEM (ii), and optical profilometry (iii) images of a 30 μm paper electrode. (b) Schematic representation of a spray-coated thick paper electrode supercapacitor. (c) CV of the thick supercapacitor recorded between 5 mV/s and 500 mV/s. (d) GCD of the supercapacitor under different current densities.

Flexible thick electrodes are potential alternatives in the field of energy storage devices to increase the energy density of devices and overcome limitations of loading of active materials and mechanical stability. However, ion penetration through the thick electrode and adhesion between current collectors and other components, especially producing them on large scales, are challenging to solve.^65^ There are a few reports showing the fabrication of thick supercapacitor electrodes (from 1 μm to 0.5 mm), mainly using sputtering, electroplating, and electropolymerization techniques, where they suffer from poor mechanical integrity and flexibility due to the brittle nature of the materials.^66,67^ Additionally, all of these techniques require time-consuming, costly infrastructures, and yet the techniques can only cover small areas (around 1 cm^2^). To overcome the limitations, printable inks containing CNF are introduced, which is a 1D nanomaterial with excellent porosity, mechanical robustness, and as a thickening agent.^12,68^ Due to better adhesion with conductive polymers and carbon particles, CNF generates a conductive path for electrons, a highly porous network for ion penetration, and mechanical robustness in the system.^25,40,68^ Together with this ink property, spray coating can be examined to efficiently achieve a 30 μm thick paper electrode directly deposited onto a substrate. Figure 5a shows a thick 30 μm electrode, where characterization reveals the smooth, low roughness paper electrode for a 2 cm by 2 cm electrode. The film was formed after 300 spraying cycles and can be easily peeled off from the carrier glass substrate to perform electrical characterizations. (Figure 5a(i)). Figure 5a(ii) demonstrates a smooth surface of the film, sheet by sheet forming of paper layers was confirmed. Optical profilometry reveals that the 30 μm film shows a low roughness (±0.44 μm) and an even surface, where good interfacial contact is required to fabricate organic electronic devices (Figure 5a(iii)). To fabricate a functional thick electrode supercapacitor, 30 μm thick electrodes were sprayed on the current collectors. The device is structured as a full-cell configuration, as shown in Figure 5b. CV and GCD tests are represented in Figure 5c,d (for clear visibility, related CVs and GCDs are provided in Figures S8 and S9). For high scan rates, even 500 mV/s, the device represents a rectangular CV with low resistive effects, exhibiting rapid charge transport. The device represents symmetrical triangular GCD curves between low (at 0.05 mA/cm^2^) and high (2.0 mA/cm^2^) current densities. The supercapacitor exhibits 9.1 mF/cm^2^ at 0.05 mA/cm^2^ (Figure S10) with a very low ESR of 0.35 Ω showing low ion diffusion resistance and good adhesion to alleviate contact issues between the layers of the supercapacitor (see Nyquist plot in Figure S11). Table S4 shows other PEDOT-based supercapacitors for comparison. The areal capacitance is at least comparable to or better than previously reported PEDOT composites, and the ESR value is quite low when it is compared with previously reported values.^37,60,69^

**Figure 6:**
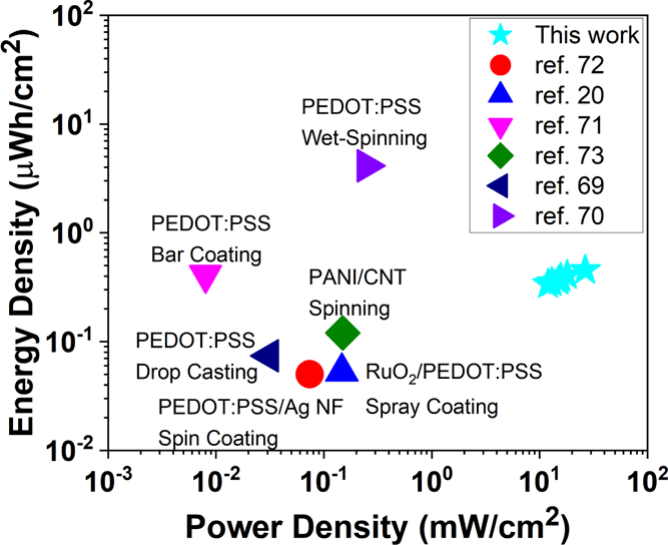
Ragone plot showing comparison for solution-processed wearable supercapacitor performance.

The measured energy and power densities of the supercapacitor are 0.453 μWh/cm^2^ and 26.5 mW/cm^2^. These values are better or comparable with the previously published PEDOT:PSS-based wearable supercapacitors, for example, RuO_2_/PEDOT:PSS spray-coated thin electrode^20^ (0.053 μWh/cm^2^, 0.147 mW/cm^2^), PEDOT:PSS coated on cloth^69^ (0.074 μWh/cm^2^, 0.031 mW/cm^2^), PEDOT:PSS fibers^70^ (4.13 μWh/cm^2^, 0.25 mW/cm^2^), and other related devices are given for comparison in a Ragone plot^71−73^ (Figure 6). The Ragone plot reveals that when the thickness is controlled, our technique brings the advantages of high power density with an area control on the device with the up-scalability property. When the total thickness of the device is considered, the device exhibits 54 μWh/cm^3^ energy and 1.78 W/cm^3^ power densities, where these values are well situated in between batteries and electrolytic capacitors (Figure S12). Additionally, the device demonstrates a reasonable self-discharge performance, 0.4 V remaining after charging to 0.6 V and self-discharged for 1 h, as shown in Figure S13.

#### 3.2.2 Influence of Spray Coating and Drop-Casting on Device Performance

The difference between the morphology and crystallinity of the paper electrodes with solution-processed-based fabrication techniques plays a vital role to understand electrode kinetics, and in Section 3.1, we have probed into the film structure and observed the difference. The most distinct quantitative result of having smaller particle formation can directly be related to a more conductive paper electrode in the case of the spray-deposited electrode (Figure S14).

To observe the effects of our findings on the device performance, a comparison was made of the electrochemical device performance of supercapacitors, which are fabricated by drop-casting and spray coating. For both device structures, 30 μm thick paper electrodes were manufactured. The drop-cast electrodes (30 ± 2 μm) were gently laminated onto current collectors, and then the gel electrolyte screen was printed as performed in the case for all printed devices. Finally, two layers are laminated. As can be seen from the CV curve, the device with the drop-cast electrode shows more resistive components by drifting away from the box-shaped CV (Figure S15). The calculated capacitance (from CV, *C* = *Q*/*VA*) difference is 9.45% in favor of the spray-coated device. From the GCD, a more considerable IR drop can be observed for the drop-cast device, where ESR is almost 4 times higher than the spray-coated one. Overall, our findings of the X-ray study are revealed at the device level, claiming that spray coating leads to a better electrode performance when it is compared with its drop-cast analogue, and the nanoscale structural difference suggests better electrochemical performance in favor of spray-coated devices.

### 3.3 Applications

**Figure 7:**
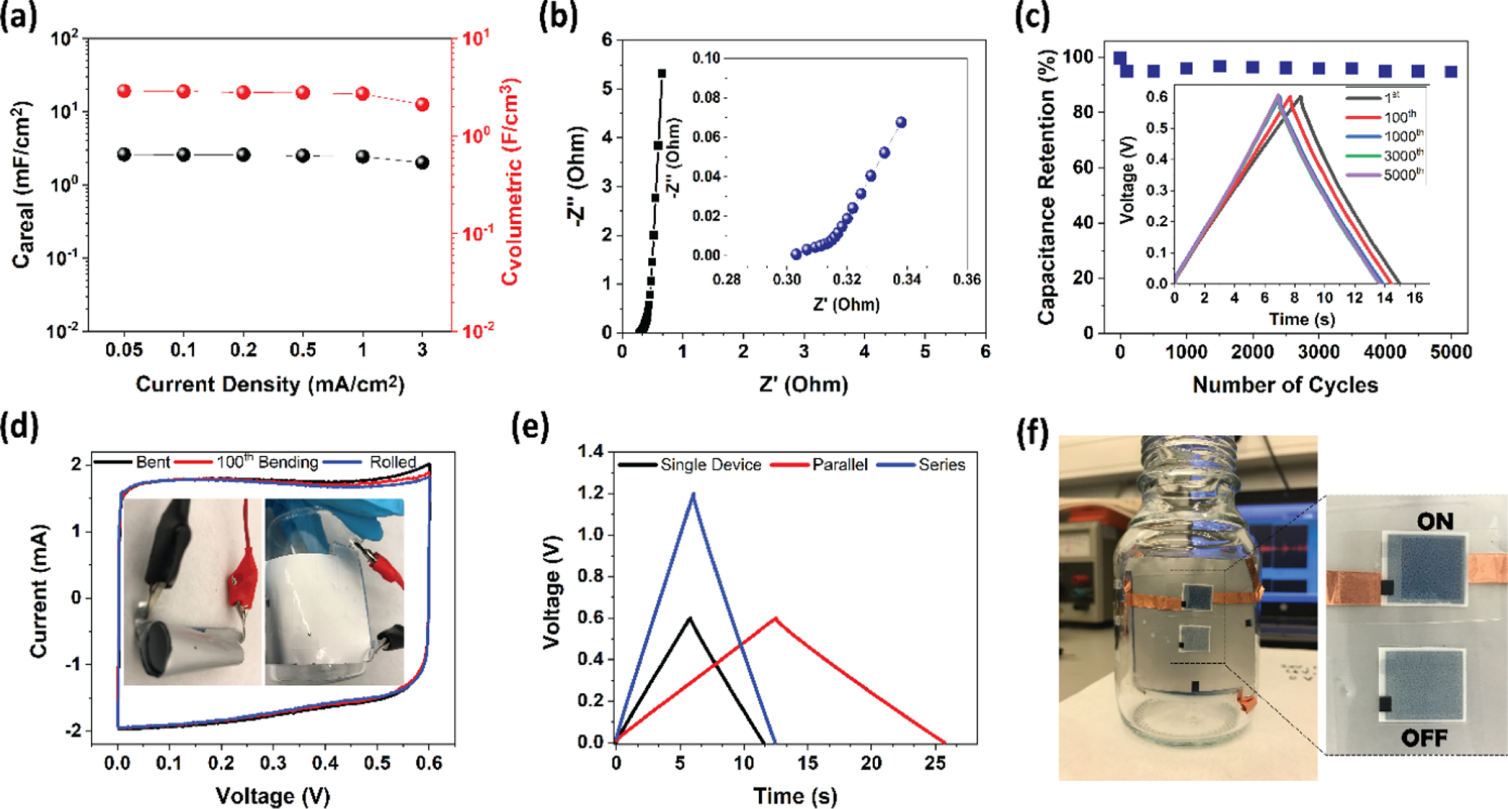
Electrochemical performance of a supercapacitor with 9 μm thick paper electrodes. (a) Areal and volumetric capacitance values calculated from GCD measurement. (b) EIS of the device between frequencies 0.02 Hz and 100 kHz, inset: zoomed image of the high-frequency area. (c) Cyclic stability of the device at a current density of 0.2 mA/cm^2^. (e) CV curve of the device under bending and rolling, inset: images of the devices under different mechanical conditions. (e, f) Application. GCD (0.2 mA/cm^2^) curves of series and parallel connected two supercapacitors, respectively. Two devices in series can supply enough charge to power an electrochromic display indicator. (f) Image of the powered electrochromic display by two supercapacitors in series, which is operated on a curved substrate.

To demonstrate possible applications and detailed electrochemical properties of the spray-coated devices, we have evaluated the device that has a 9 μm paper electrode, and the complete characterization is given in Figure 7. Typical CVs are recorded from 5 to 100 mV/s for a printed device; see Figure S15a. The GCD curves show linear, triangle shapes at different current densities, where the excellent capacitive performance of the paper device is extracted (Figure S16b). Up to 2.60 mF/cm^2^ (areal) and 2.89 F/cm^3^ (volumetric) capacitance values for different current densities are then calculated from the GCD test and represented in Figure 7a. The areal capacitance demonstrates stable capacitance values at different current rates that point to the operational capability of the devices, even low current energy harvesting technologies or from high current sources.

Since the energy density is dependent on the square of the operating voltage, increasing the charging voltage leads to higher energy densities (eq 4); however, it also causes a high IR drop and a lower Coulombic efficiency in PEDOT-based devices^69^ (Figure S17). The highest energy density of the supercapacitor is found to be 0.13 μWh/cm^2^ areal and 8.76 μWh/cm^3^, whereas outstanding power densities are 11 mW/cm^2^ and 0.75 W/cm^3^, when the total device volume is considered, as shown in Figure S18. The maximum energy and power densities that the paper electrode can deliver are 0.293 mWh/cm^3^ and 17.58 W/cm^3^, when only the paper electrode volume is considered. Furthermore, to compare with the paper-based supercapacitors, a Ragone plot is given in Figure S19. These values are comparable in terms of energy density and better in power density when they are compared with paper, and PEDOT composite-based reported supercapacitors, where our method brings quite high power together with a thin and packed device architecture.^35,60,74^ To further prove the low ESR performance of the spray-coated supercapacitor, we have performed electrochemical impedance spectroscopy (EIS) between frequencies 20 mHz and 100 kHz, see Figure 7b. The supercapacitor exhibits ∼0.3 Ω at the 100 kHz intercept of the *Z*′ in the Nyquist plot. The ESR value (0.3 Ω or 0.0148 Ω/cm^2^) is far better than the other PEDOT-based composites, mechanically strengthened supercapacitors such as the aramid nanofibers/PEDOT:PSS (7.3 Ω/cm^2^), nanocellulose/PEDOT (1.7 Ω), paper/PEDOT (6.5 Ω), and PEDOT/PEDOT:PSS/paper (6 Ω) and comparable with our previous work by airbrush-deposited PEDOT/CNF (0.140 Ω/cm^2^) and screen-printed pulp/PEDOT (0.61 Ω).^10,28,37,60,75^ Ideal capacitive response together with low resistance values is observed at low frequencies, which is quite typical of conductive polymer supercapacitors. The printing/coating technique to fabricate supercapacitors presented here is also quite reproducible and enables to achieve high yield fabrication (Table S6).

Another critical parameter to investigate the scaling up of the devices is cycling stability, especially long term. Figure 7c demonstrates excellent capacitance retention of the device, with 95% of the initial capacitance of the device maintained even after 5000 cycles with a Coulombic efficiency of 94%, showing stability at long-term operation. To demonstrate the mechanical robustness of the printed devices, voltammograms are recorded under different conditions such as when the device is bent, rolled, and after bending 100 times, see Figure 7d. The device operates well at a bending radius (*R* = 0.6 cm) during the 100th cycle (supporting information, Figure S20). The paper network improves mechanical durability, and during the bending test, it provides good mechanical stability with negligible electrochemical performance loss. The CV curves at a 20 mV/s scan rate demonstrate almost the same electrochemical performance under mechanical tests, proving that the spray-coated supercapacitors are durable and readily available to power up low-power organic electronics and to be integrated with wearable electronics. Since our devices exhibit excellent cyclic stability, mechanical robustness, provide an mF range of capacitance, an application is demonstrated in Figure 7e–f. To demonstrate a practical application, two printed supercapacitors are connected in series to increase the voltage window, and voltammograms at 100 mV/s show quite stable and rectangular CVs, indicating a minor charge balance issue, see Figure S21. The tandem device represents a potential window of 1.2 V (Figure 7e), which is adequate to supply a low-power (1–1.5 V) electrochromic indicator. Figure 7f shows the GCD (0.2 mA/cm^2^) of serially connected supercapacitors placed on a curved surface to demonstrate operation on unconventional substrates. During the operation, two serially connected supercapacitors are charged up to 1.2 V and then connected to an electrochromic display; as can be seen (Figure 7f, inset), it is quite enough and stably powers an electrochromic indicator.

### 3.4 Integrated Wearable Power Unit

**Figure 8:**
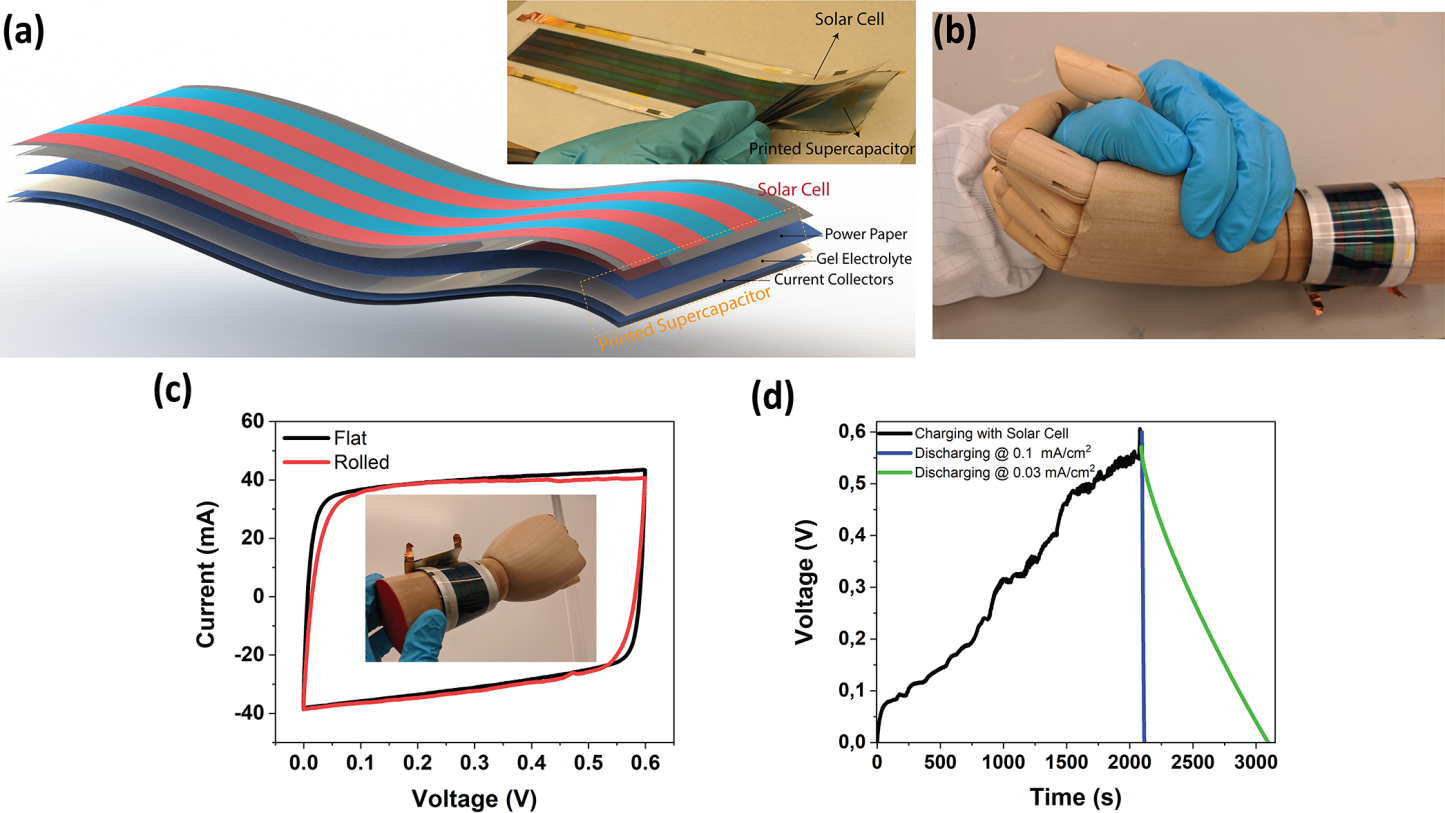
Wearable technology combination. (a) Schematic representation of a large-area flexible solar cell for indoor applications and printed paper supercapacitors. (b) Image of an integrated wristband on a model hand. (c) CV (100 mV/s) curves of a large-area supercapacitor when it is applied as a wristband. (d) Charging curve of the supercapacitor with solar cells (indoor lightning) and discharging curves of the printed supercapacitor at a current density of 0.03 and 0.1 mA/cm^2^.

Balancing energy fluctuations, rapid power delivery, and storing charge from low current sources are needed in self-sustaining technologies. Supercapacitors are efficient and low-cost energy storage devices to power consumer electronics, displays, and low-power systems.^10,69,76^ Especially using waste energy from indoor lighting, balancing light fluctuations becomes another essential aspect of green energy technology.^77^ Combining clean energy sources with flexible supercapacitors provides a prominent solution to develop smart IoT systems and self-powered wearable electronics. The need for energy packs for both printed and textile-based devices is increasing rapidly to boost supercapacitor research to advance in wearable technologies.^78,79^ As illustrated in Figure 8a, a large-area printed supercapacitor is combined with a flexible indoor solar cell module and assembled as a wearable power package. To provide high capacity and demonstrate the scalability of spray-coated paper supercapacitors, we have fabricated a 5 cm by 18 cm large-area device (with *a* ≈ 10 μm thick paper electrode) and a fully printed, wearable device is assembled (Figure S22). The wearable supercapacitor demonstrates typical CV curves with small resistive components (Figure S23). The GCD test supplies ≈0.5 F at 0.3 mA/cm^2^, see Figure S24. Figure 8b demonstrates the CV of the device when it is rolled or flat, which illustrates that electrochemical performance is stable when the device is rolled (as wrist-worn). The overall thickness of the supercapacitor is 180 μm, and its mechanical form factor is quite good and can be rolled during the operation. The designed integrated system has an overall thickness of 0.38 mm and can be worn (model in Figure 8b). The solar unit photocharges the supercapacitor up to 0.6 V; then discharging can be started with a little bit onset of discharge, see Figure 8d. The solar cell can supply enough power to charge the supercapacitor under ambient indoor lighting conditions (open-circuit voltage of 3.6 V, Figure S25). The charging from the solar cell (under indoor light) and discharging at constant current were recorded. Unlike the case for solar simulators, indoor light conditions fluctuate continuously, and it should be noted that the charging time takes a bit longer due to the interrupted light intensity. An efficiency of 2.7% was recorded from the flexible self-powered pack, which is comparable to recently published entirely flexible solar cell/supercapacitor systems.^7,76,80^ Even though this value is comparably low, it should be noted that this integration is the first-generation demonstrator of harvesting such waste energy from indoor lightning and using the concept as a part of a self-sustained wearable package. With such wearable and well-packed energy systems, we envision that solar cell modules, which can harvest waste light from indoors and combine with large-scale paper supercapacitors as a storage unit, are excellent candidates for wearable self-powered systems.

## 4 Conclusions

In this work, we demonstrate a paper-based supercapacitor integrated in a wearable device with superior electrochemical performance. We present and verify a versatile fabrication route for printed paper-based supercapacitors toward scaling up the design of large-area devices for flexible and wearable organic electronics. Paper electrodes were fabricated with thicknesses from 1.7 to 30 μm to demonstrate spray deposition capability to achieve large area, thick paper electrodes, which are readily applied in a wearable device. Optimum performance is found when employing spray coating. Due to its nonequilibrium process parameters, it leads to favorable, smaller conductive polymer feature sizes, more homogeneous coating, and pronounced crystallinity, being beneficial to achieve excellent contact with device layers and leading to low ESR values in the composite system when compared to the traditional electrode-making process (drop-casting). The stability of the spray-coating approach is proven with 95% capacitance retention after 5000 cycles and bending tests. The all-spray deposited/printed supercapacitor exhibits excellent electrochemical and mechanical performance and supplies enough energy to power printed electrochromic displays. Because of all sequentially coated/printed fashion, our method proposes a low-cost, R2R compatibility to fabricate sustainable wearable devices. Moreover, the large-area supercapacitor is integrated into a flexible solar cell module to demonstrate a self-powered wristband, which expands the way for printing technologies to achieve low-cost, scalable, and self-sustaining energy systems to be used in robotics, prosthetics, and health trackers.

## Supporting Information Available

GISAXS analysis; nanostructural parameters obtained from GISAXS analysis using one-dimensional fit with the Porod–Guinier model (Table S1); comparison of conductivity values of different flexible electrodes (Table S2); viscosity of the paper electrode ink as a function of the shear rate and contact angle of the power paper ink on the carbon adhesion layer (Figure S1); and AFM topography image of the drop-cast paper electrode (Figure S2) (PDF)
