# Supplementary material for: Scalable Paper Supercapacitors for Printed Wearable Electronics
Source: ACS Appl Mater Interfaces. 2022 Dec 12;14(50):55850–63. doi: 10.1021/acsami.2c15514 (PMC9782359; doi:10.1021/acsami.2c15514)
Supplement: Supplementary file 1 — am2c15514_si_001.pdf [file am2c15514_si_001.pdf]

Supporting Information

Scalable Paper Supercapacitors for Printed Wearable Electronics

Mehmet Girayhan Say[a], Calvin J. Brett[b,c,d], Jesper Edberg[e], Stephan V. Roth[d,f], Daniel Söderberg[b,c], Isak Engquist[a,g,*], Magnus Berggren[a,g]

*Corresponding author: isak.engquist@liu.se

[a]Laboratory of Organic Electronics, Department of Science and Technology, Linköping University, SE-601 74 Norrköping, Sweden

[b]Wallenberg Wood Science Center, KTH Royal Institute of Technology, Teknikringen 56-58, 100 44 Stockholm, Sweden

[c]Department of Engineering Mechanics, KTH Royal Institute of Technology, Osquars Backe 18, 100 44, Stockholm, Sweden

[d]Deutsches Elektronen-Synchrotron (DESY), Notkestrasse 85, 22607 Hamburg, Germany

[e] RISE Research Institutes of Sweden, Digital Systems, Bio- and Organic Electronics, Bredgatan 35, Norrköping SE-602 21, Sweden

[f]Fibre and Polymer Technology, KTH Royal Institute of Technology, Teknikringen 56-58, 100 44 Stockholm, Sweden

[g]Wallenberg Wood Science Center, ITN, Linköping University, SE-601 74 Norrköping, Sweden

GISAXS Analysis:

After Hammouda[1] s =1 defines rods with a cross-sectional radius of $R = R_G \cdot \sqrt{2}$ and s = 0 defines spherical features with a radius of $R = \frac{R_G}{\sqrt{3/5}}$. The Porod exponent p = 2 results from Gaussian polymer chains and p = 5/3 from "fully swollen" polymer chains.

Table S1. Nanostructural parameter obtained from GISAXS analysis using one-dimensional fit with Porod-Guinier model.

	R_{G1} [nm]<br>$R_{sph.}$ [nm]	s_1	p_1	R_{G2} [nm]<br>$R_{cyl.}$ [nm]	s_2	p_2
Spray deposited	37.1 ± 0.1<br>**47.9 ± 0.1**	0.27 ± 0.03	2.3 ± 0.2	2.1 ± 0.2<br>**1.6 ± 0.3**	0.96 ± 0.02	1.7 ± 0.3
Drop casted	59.9 ± 0.1<br>**77.3 ± 0.1**	0.25 ± 0.02	2.2 ± 0.1			

Table S2. Comparison of conductivity values of different flexible electrodes.

Electrode	Conductivity	Reference
**PEDOT:PSS/CNF**	112 S/cm	This work
**PEDOT/paper**	375 S/cm	2
**PEDOT/ PEDOT:PSS/ paper**	1500 S/cm	3
**PEDOT nanofibers**	3580 S/cm	4
**Mxene**	15100 S/cm	5
**CNT**	624 S/cm	6
**PEDOT:PSS/Graphene**	1000 S/cm	7
**Aramid NF/PEDOT:PSS**	534 S/cm	8
**RGO/MnO_2**	532 S/m	9
**Graphene Paper**	1700 S/m	10

Note on conductivity:

The sheet resistance and conductivity of the thin paper electrodes can be calculated using the following equations:

$$R_S = 4.53\frac{\Delta V}{I} \text{ and } \sigma = \frac{1}{R_S t}$$

Where R_S is the conductivity, ΔV is the change in voltage measured between the inner probes, I is the current applied between the outer probes in the four point probe system. σ is the conductivity, t is the thickness of the electrodes.

Table S3. Comparison table for ESR values of the supercapacitors with different electrode thickness at current density of j = 0.2 mA/cm^2.

Thickness (μm)	ESR (Ω)	ESR (Ω cm^2)
1.7 ±0.07	0.49	9.8
3.0 ±0.12	0.45	9
6.2 ±0.2	0.6	12
9 ±0.25	0.4 ±0.03	8
20 ±0.5	0.37	7.4
30 ±0.44	0.35	7

Table S4. Comparison of performance of PEDOT based supercapacitors.

Electrode	Electrode Thickness	Capacitance	ESR	Reference
**PEDOT:PSS /Al**	220 nm	51 μF	<1 Ω	11
**PEDOT:PSS/Ag Grid**	<1 μm	7.36 mF/cm^2	-	12
**PEDOT:PSS Fiber**	d= 60 μm	119 mF/cm^2	100 Ω/cm	13
**PEDOT:PSS/AgNF**	90 nm	0.91 mF/cm^2	33.1 Ω	14
**PEDOT coated Fabric**	-	0.64 mF/cm^2	1<R<10	15
**PEDOT:PSS**	<1 μm	4.72 mF/cm^2	-	16
**PEDOT:PSS/Aramid Fibers**	14.8 μm	111.5 F/g	7.3 Ω	17
**PEDOT:PSS**	300 nm	994 μF/cm^2	0.09 Ω/cm^2	18
**PEDOT:PSS/H_2SO_4**	2.78 μm	50.1 F/cm^3	5.7 Ω	19
**PEDOT:PSS coated textile**	-	10 mF/cm^2	6.3 Ω	20
**PEDOT Paper**	8 μm	115 mF/cm^2	6.5 Ω	2
**PEDOT Paper**	200 μm	920 mF/cm^2	1.7 Ω	21
**PEDOT:PSS/Pulp**	100 μm	9.2 mF/cm^2	0.61 Ω	22

Table S5. Specific capacitance comparison table

Electrode	Specific Capacitance	Reference
**PEDOT:PSS/CNF**	26 F/g	This work
**PEDOT/paper**	20 F/g	2
**PEDOT/ PEDOT:PSS/ paper**	101 F/g	3
**VPP PEDOT/Cellulose Paper**	24 F/g	23
**PEDOT nanopaper**	90 F/g	24
**MWCNT/PEDOT:PSS/cellulose**	140 F/g	25
**PANI/Bacterial cellulose/Graphene**	477 F/g	26
**Mxene/Nanocellulose**	211 F/g	27

Table S6. Number of supercapacitors with 9 µm electrode, showing high yield of fabrication. Data calculated for 0.2 mA/cm^2 current density.

Device Number	Areal Capacitance (mF/cm^2)	ESR (Ω)
1	2.56	0.4
2	2.29	0.37
3	2.2	0.3
4	2.54	1

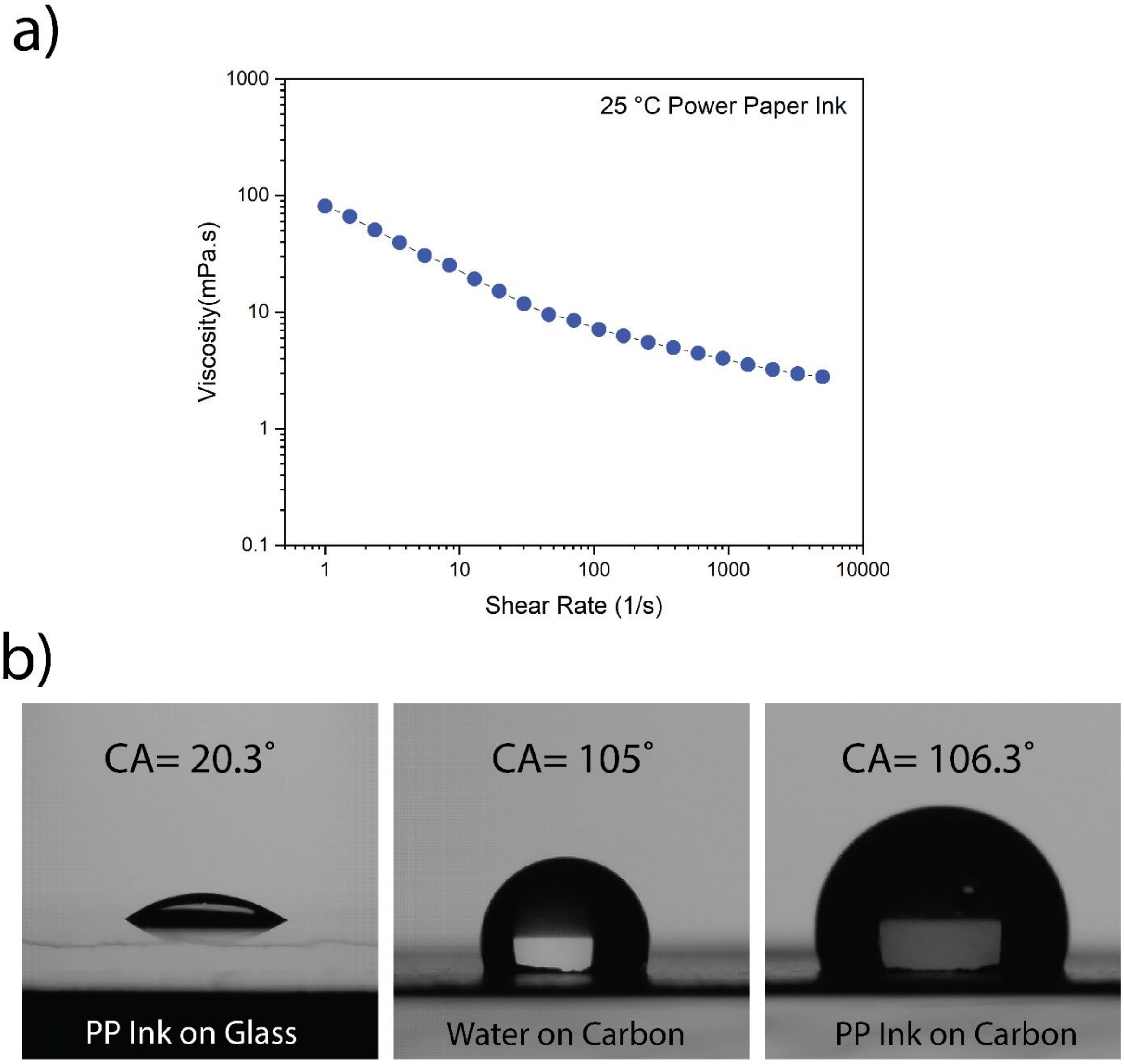

Figure S1. a) Viscosity of the paper electrode ink as a function of shear rate. b) Contact angle of power paper ink on carbon adhesion layer.

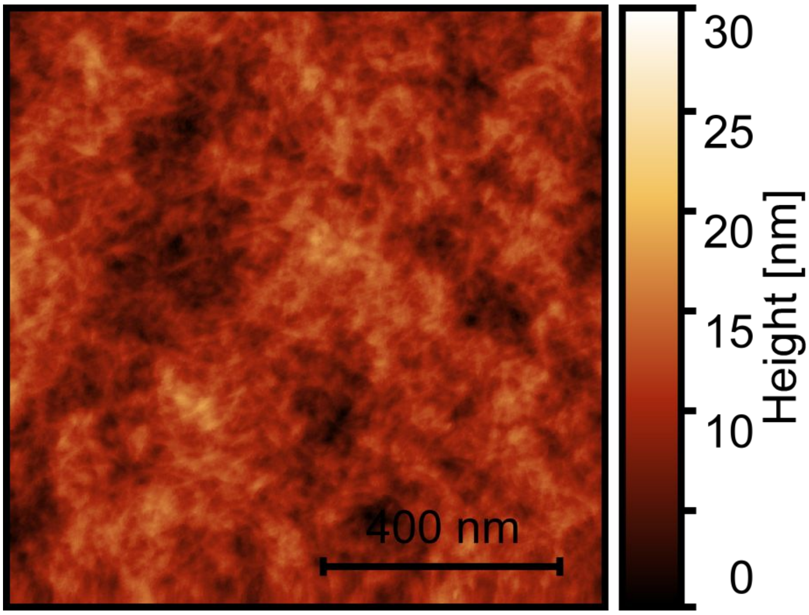

Figure S2. AFM topography image of the drop-casted paper electrode.

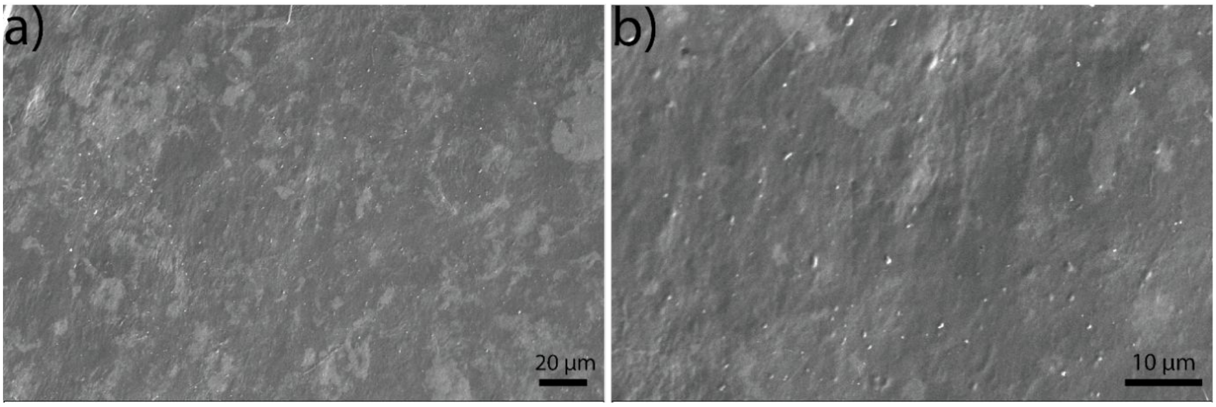

Figure S3. SEM images of 30 µm paper electrode. b) Zoomed in image of a).

a)

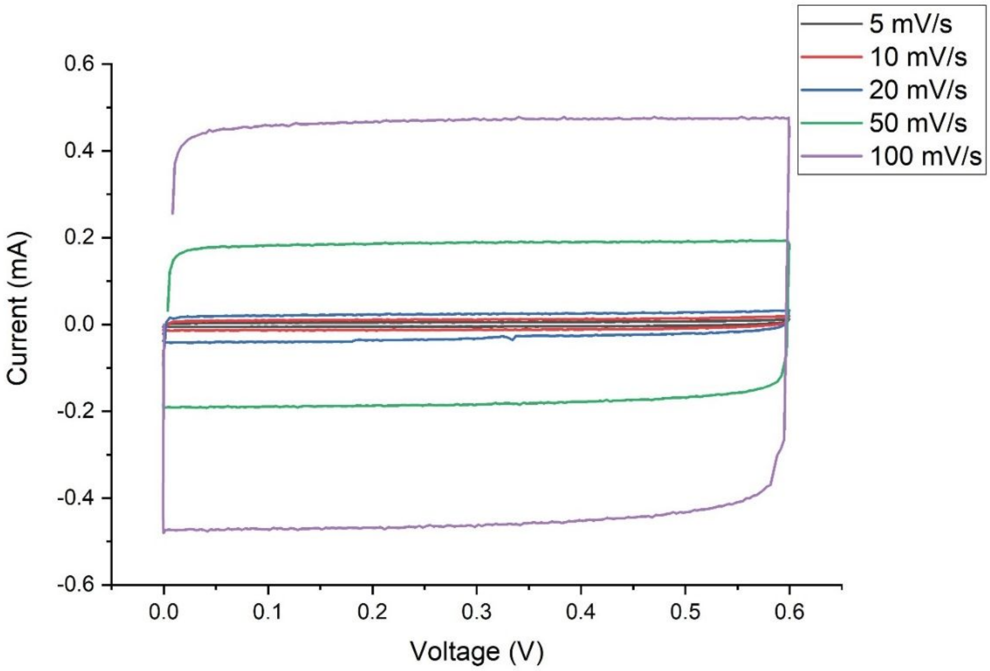

b)

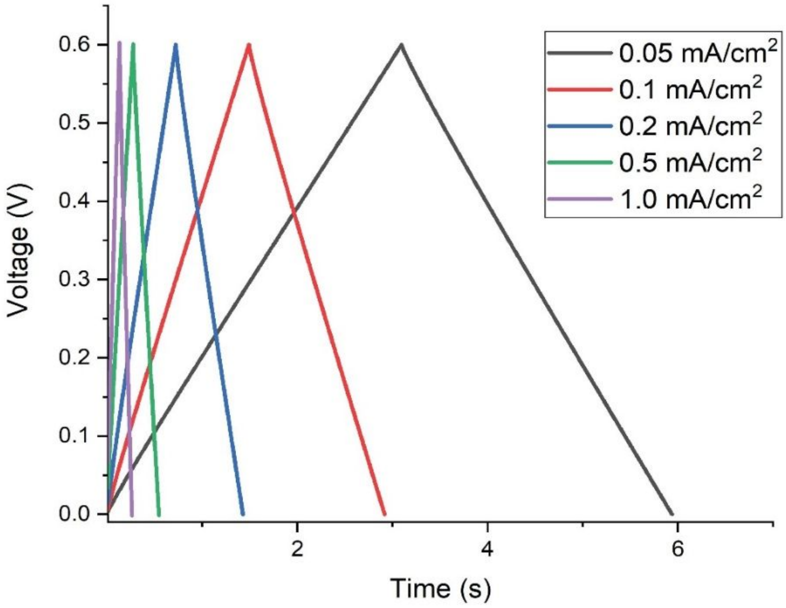

Figure S4. a) CV and b) GCD of 15x (1.7 μm) Supercapacitors.

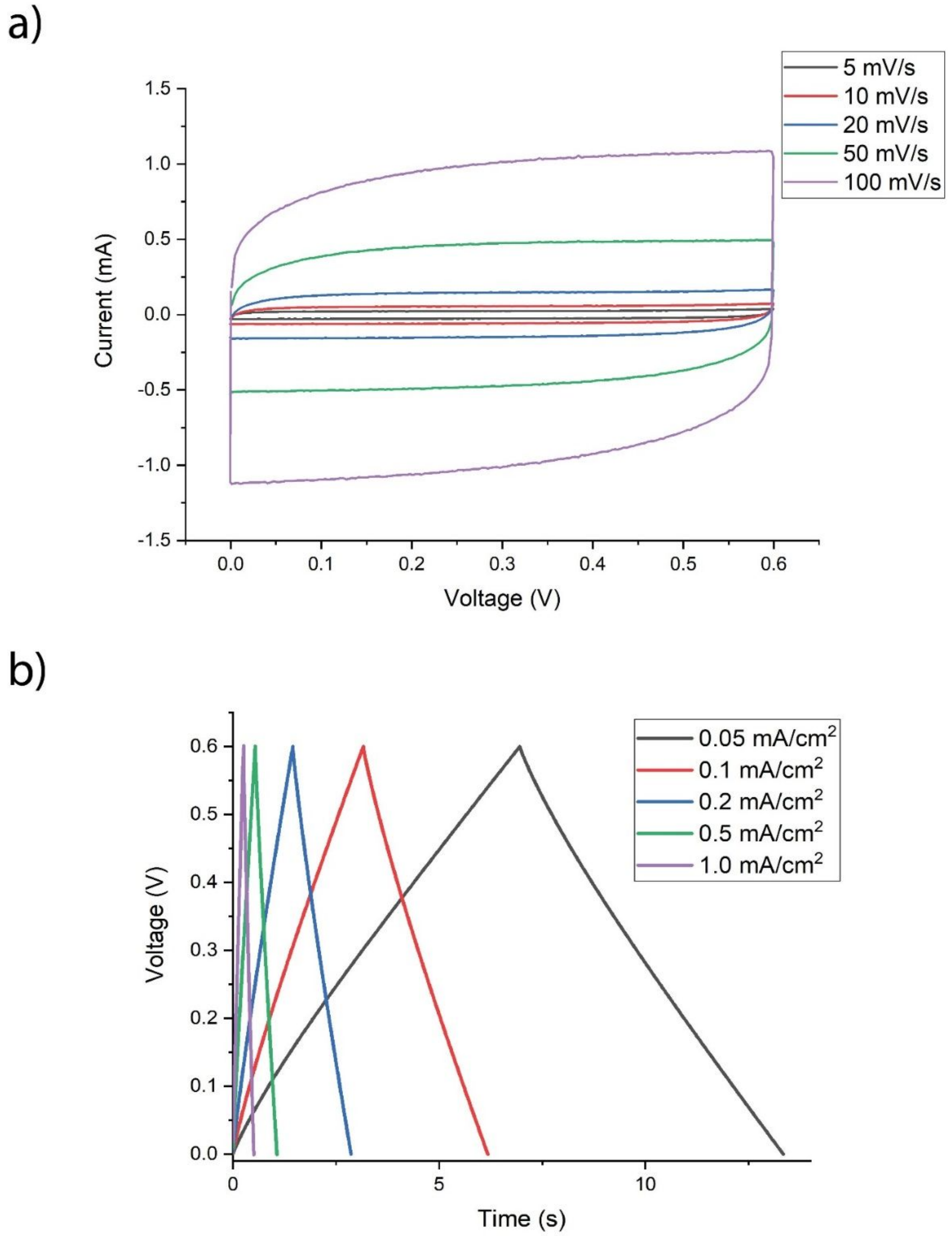

Figure S5. a) CV and b) GCD of 30 x (3.0 µm) Supercapacitors.

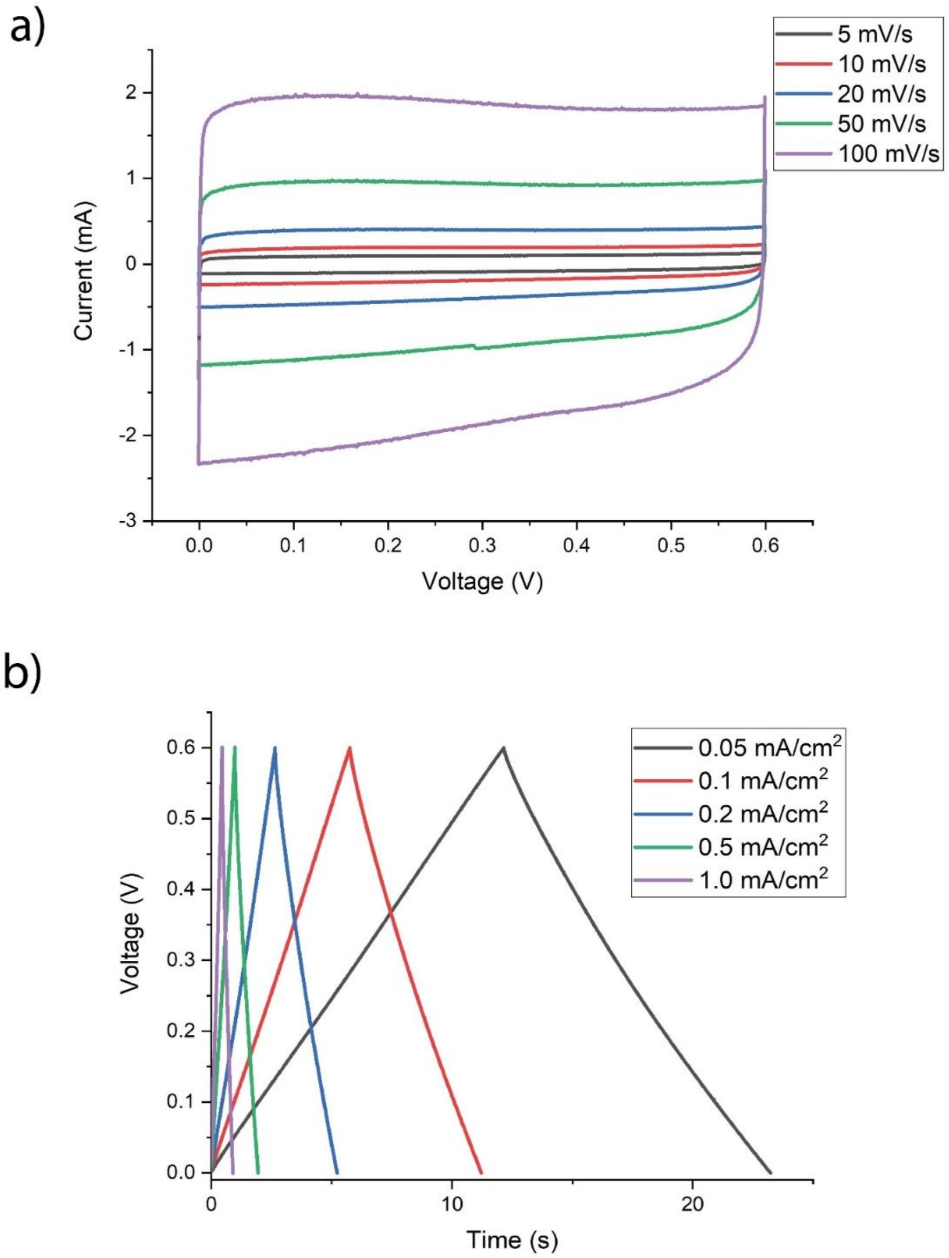

Figure S6. a) CV and b) GCD of 60 x (6.2 μm) Supercapacitors.

a)

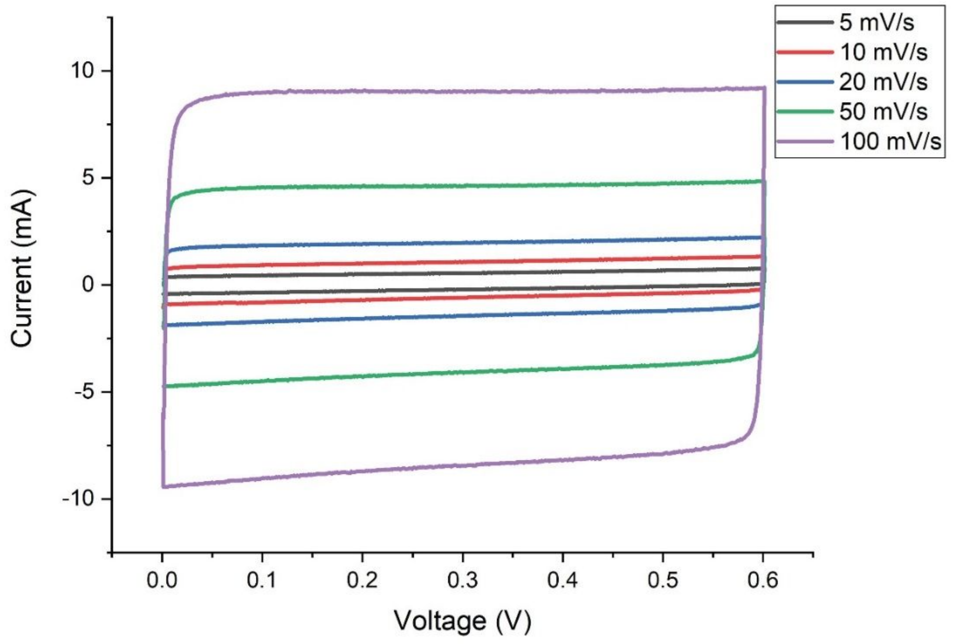

b)

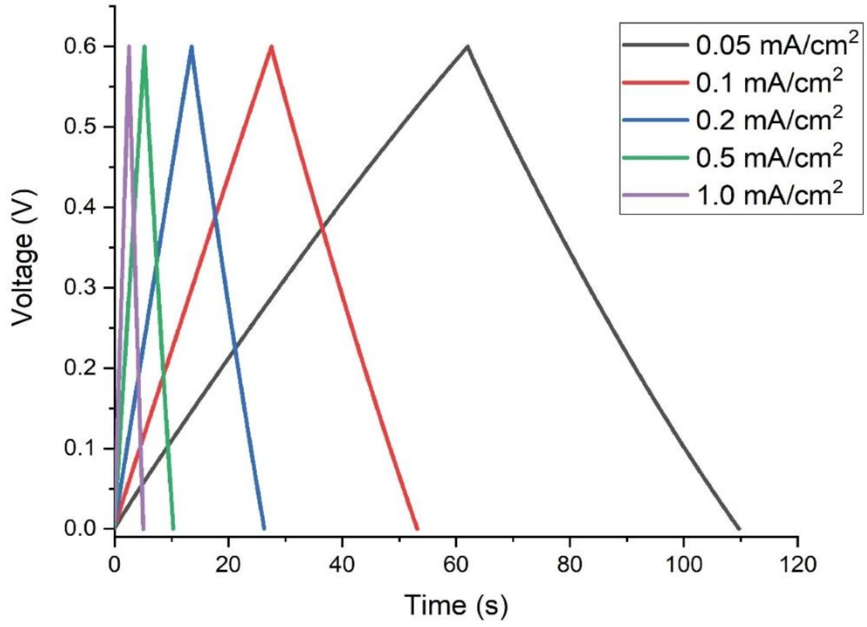

Figure S7. a) CV and b) GCD of 180 x (20 µm) Supercapacitors.

a)

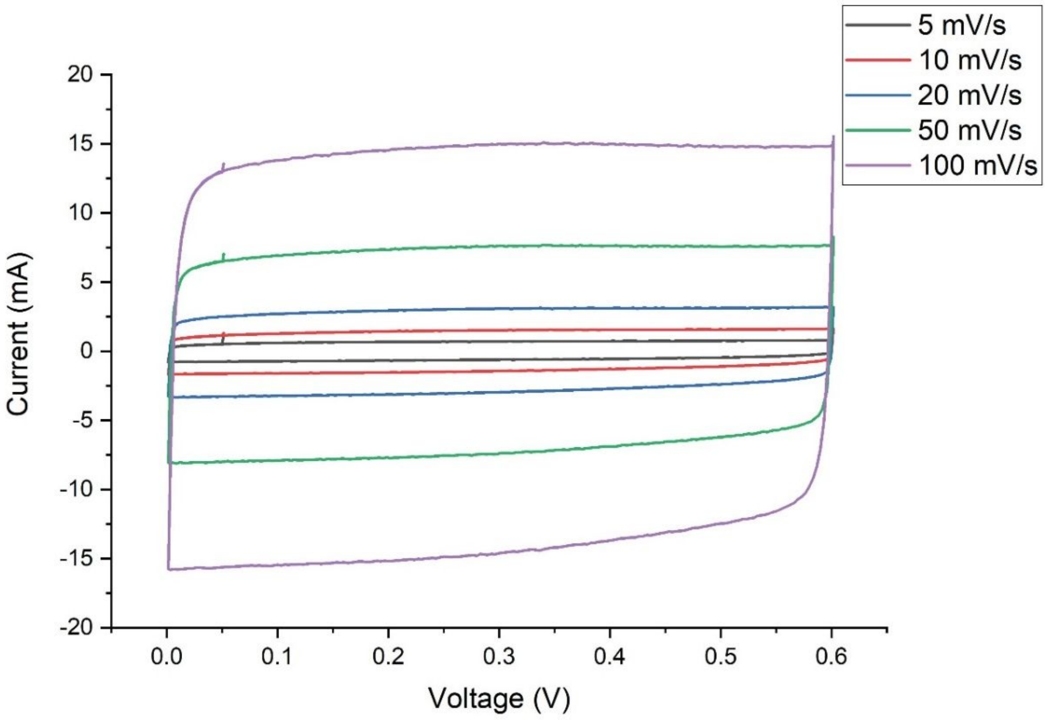

b)

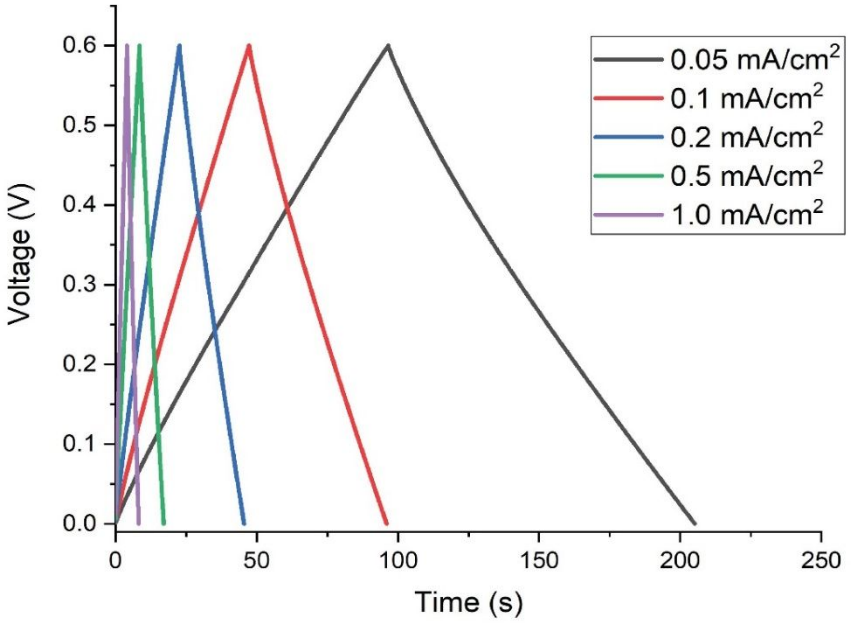

Figure S8. a) CV and b) GCD of 300x (30 µm) Supercapacitors.

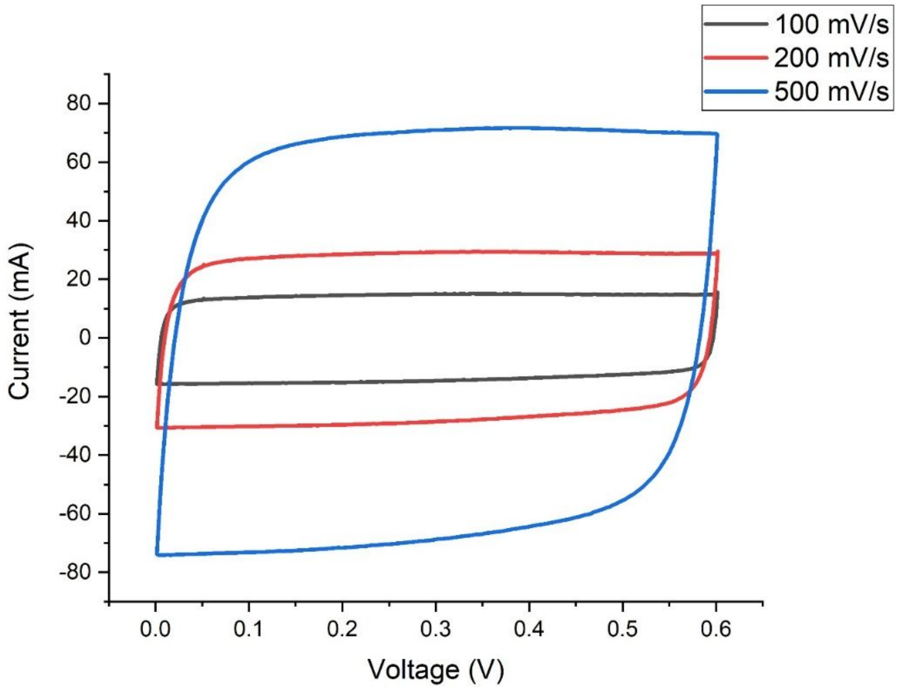

Figure S9. a) CV of 30 μm thick electrode at higher scan rates (100, 200, 500 mV/s).

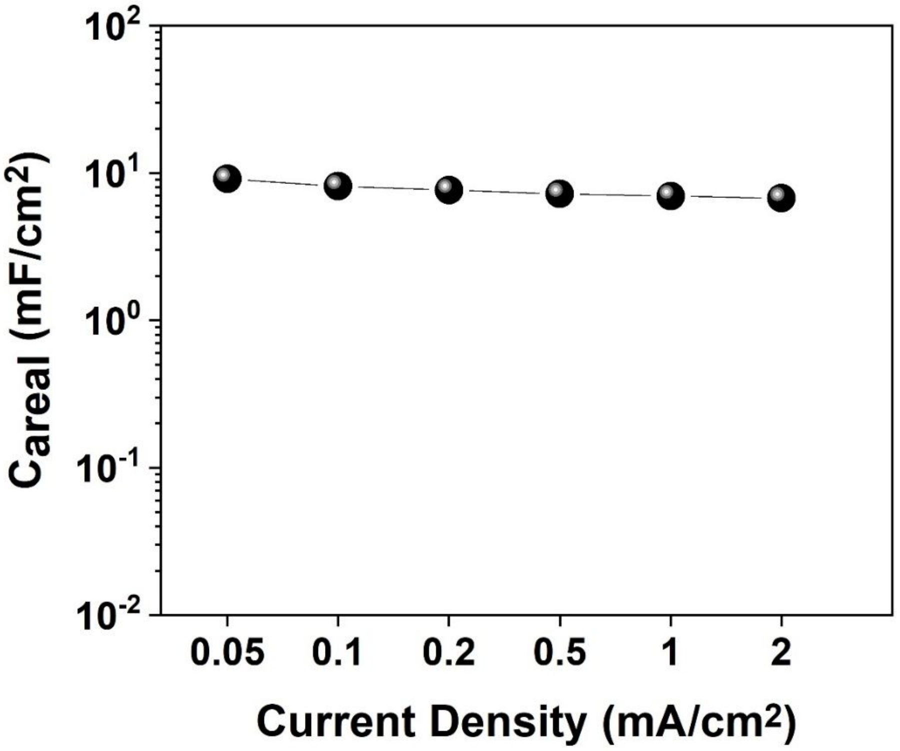

Figure S10. Areal capacitance vs. current density for supercapacitor with electrode thickness of 30 μm electrode.

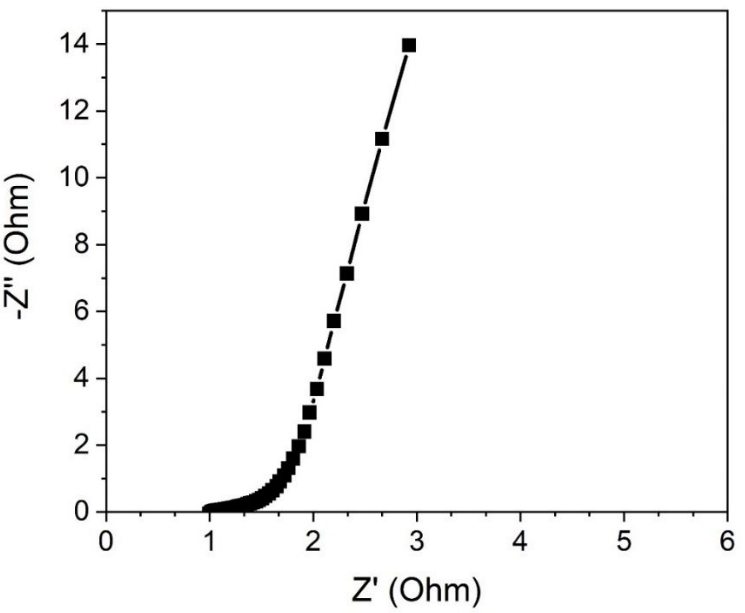

Figure S11. Nyquist plot supercapacitor with 30 μm electrode.

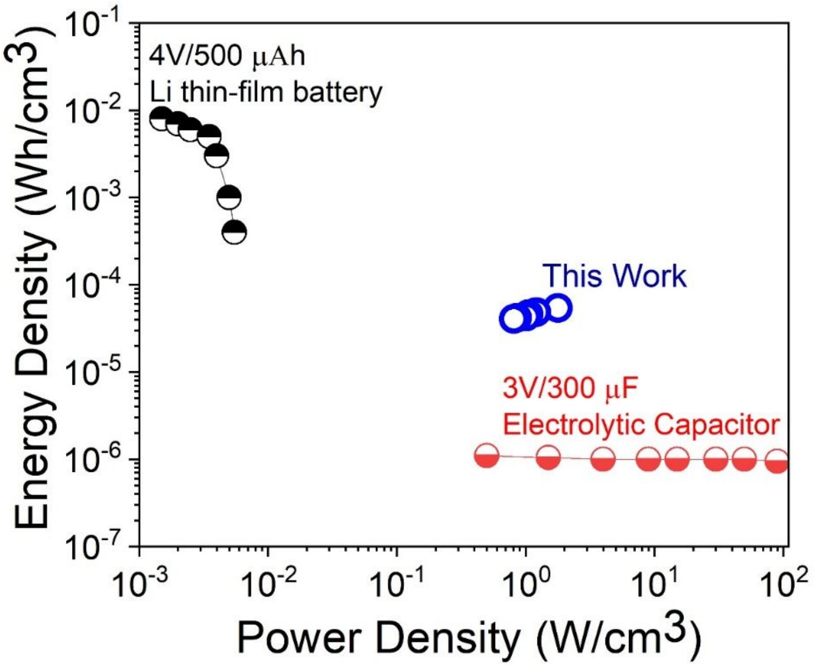

Figure S12. Ragone Plot for comparison of paper supercapacitors with commercial Li battery and Electrolytic capacitors.

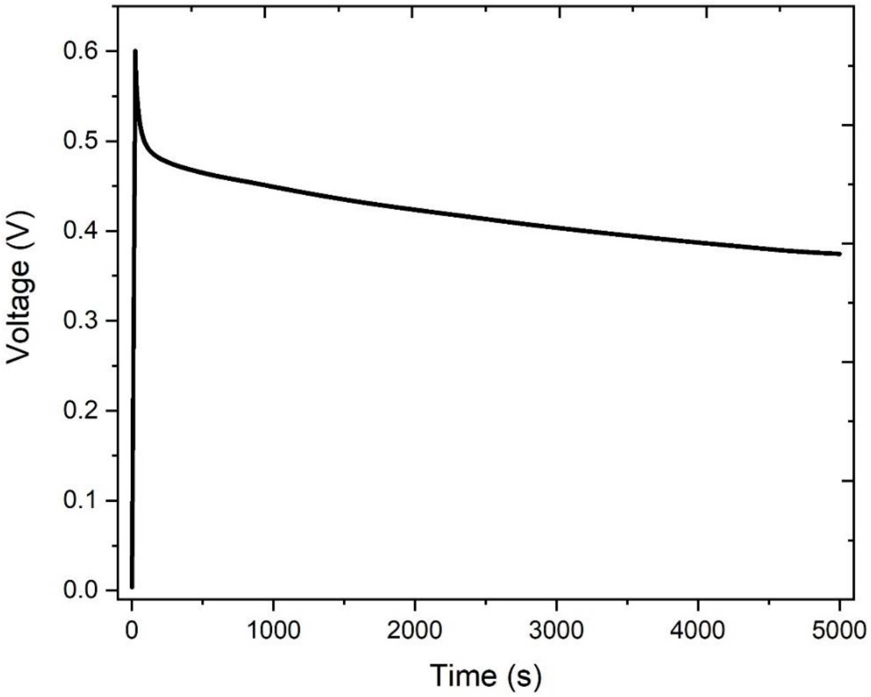

Figure S13. Self-Discharge Performance of a supercapacitor with 30 µm thick electrode. The device is charged with a current density of j = 0.2 mA/cm^2.

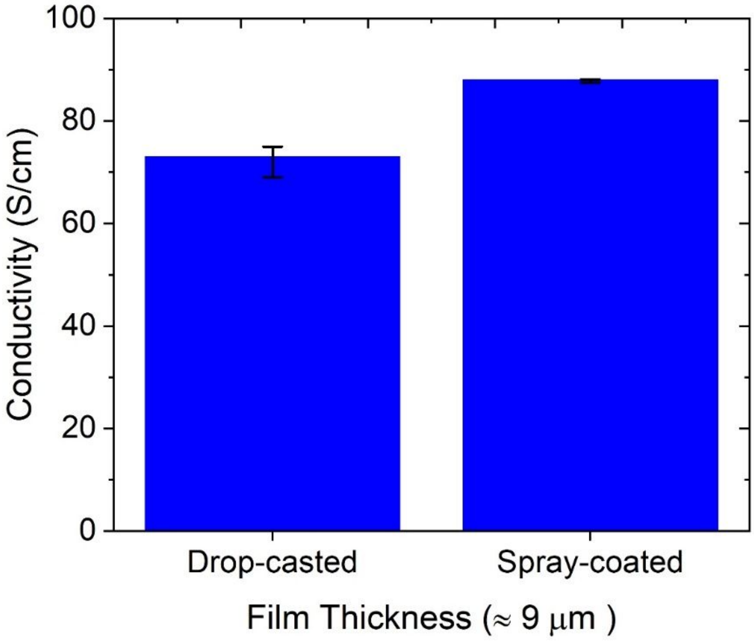

Figure S14. Electrical conductivity comparison for drop-casted and spray coated paper electrodes.

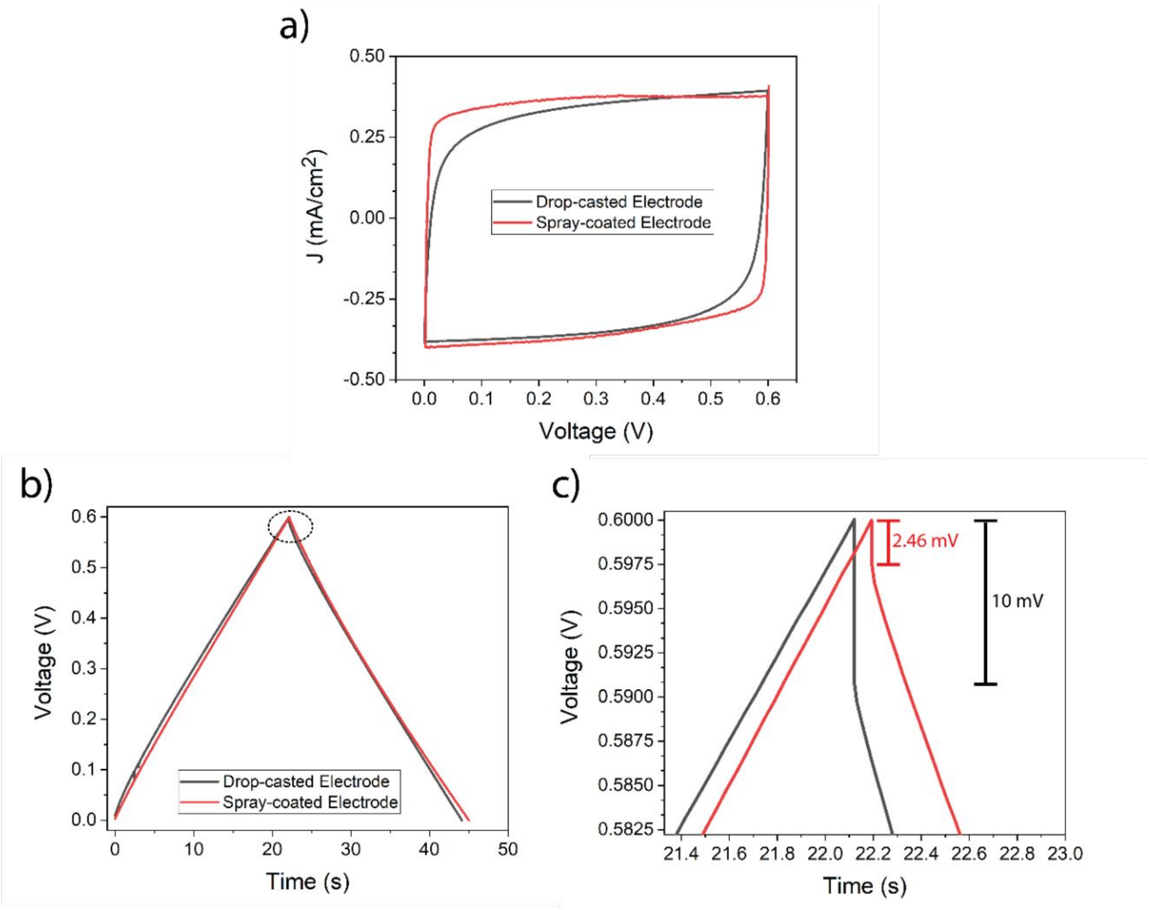

Figure S15. Electrochemical device performance comparison for 30 μm drop casted and spray coated electrode. a) CV at 50 mV/s. b) GCD at 0.2 mA/cm^2. c) Closer look to dashed circle at (b) to evaluate IR drop.

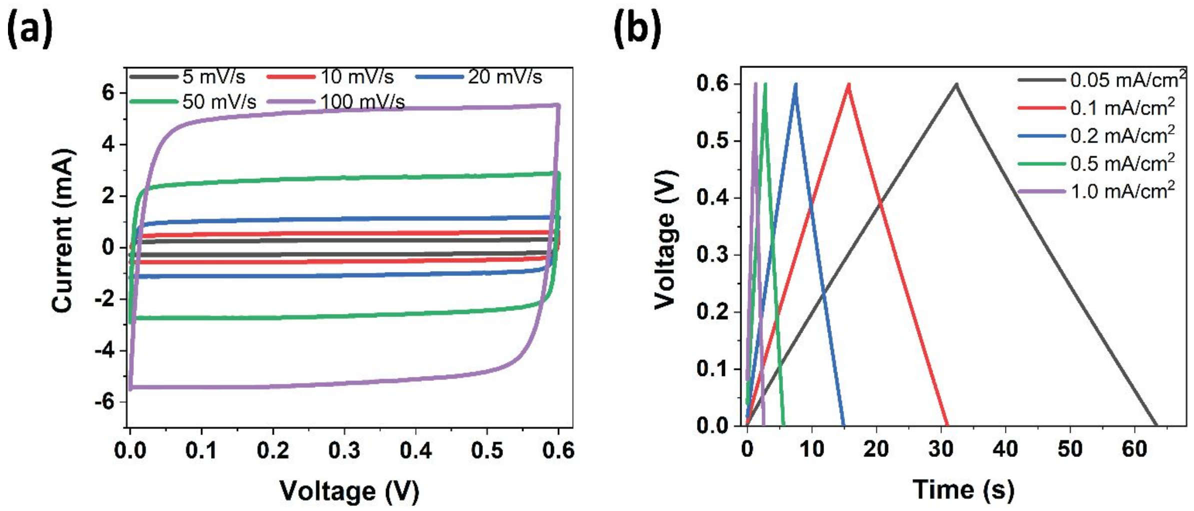

Figure S16. a) CV curves of supercapacitor with 9 µm paper electrodes recorded at scan rates from 5 mV/s to 100 mV/s. b) Galvanostatic charge-discharge curves at a discharge current of 0.05, 0.1, 0.2, 0.5 and 1.0 mA/cm^2.

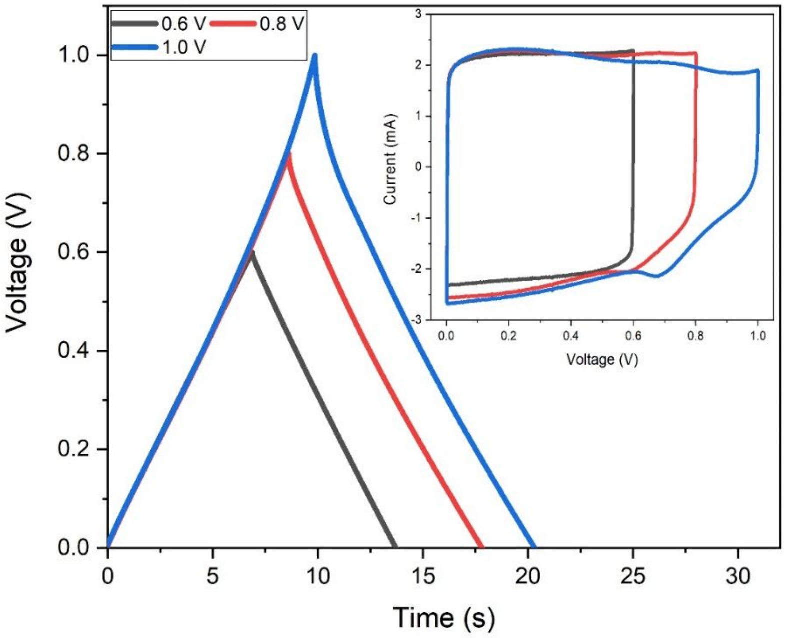

Figure S17. Safe operation voltage for power paper supercapacitors. GCD of the devices at 0.2 mA/cm^2. Inset, CV of the device at 100 mV/s for device that has 9 µm electrode.

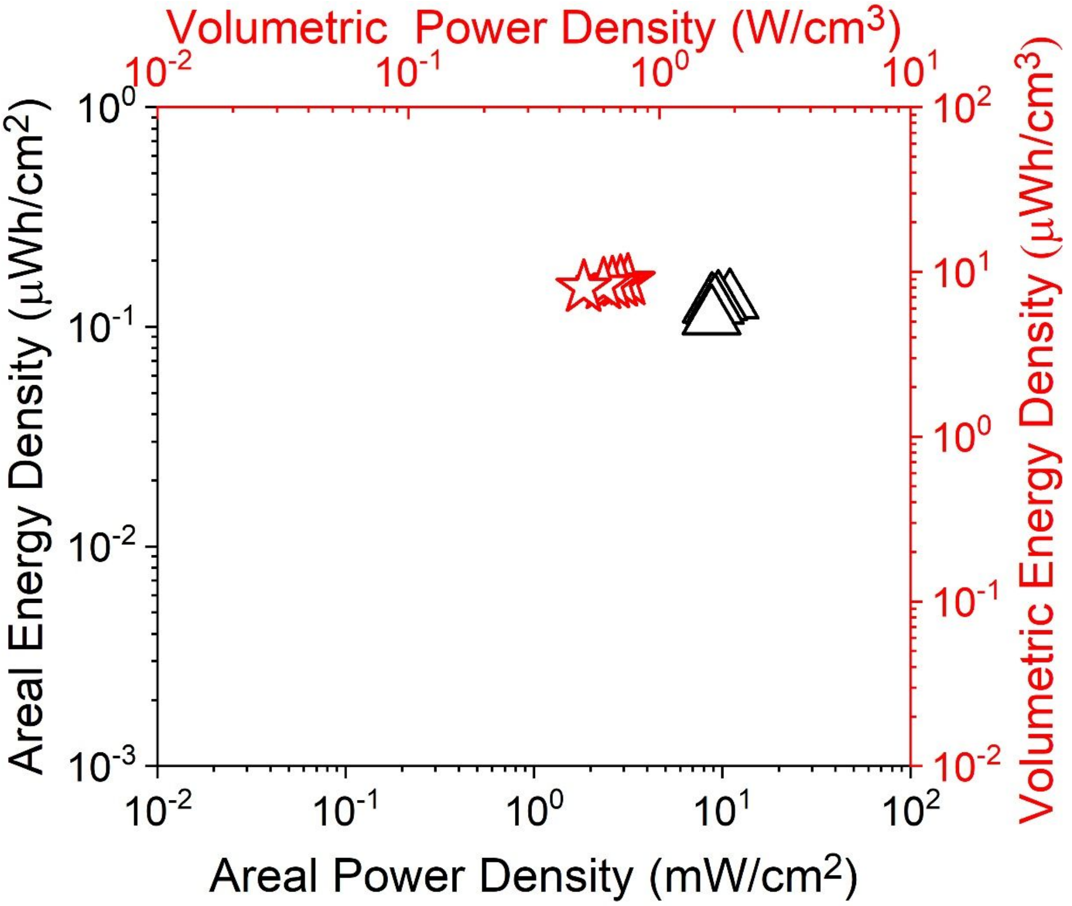

Figure S18. Ragone plot for supercapacitor with electrode thickness of 9 μm.

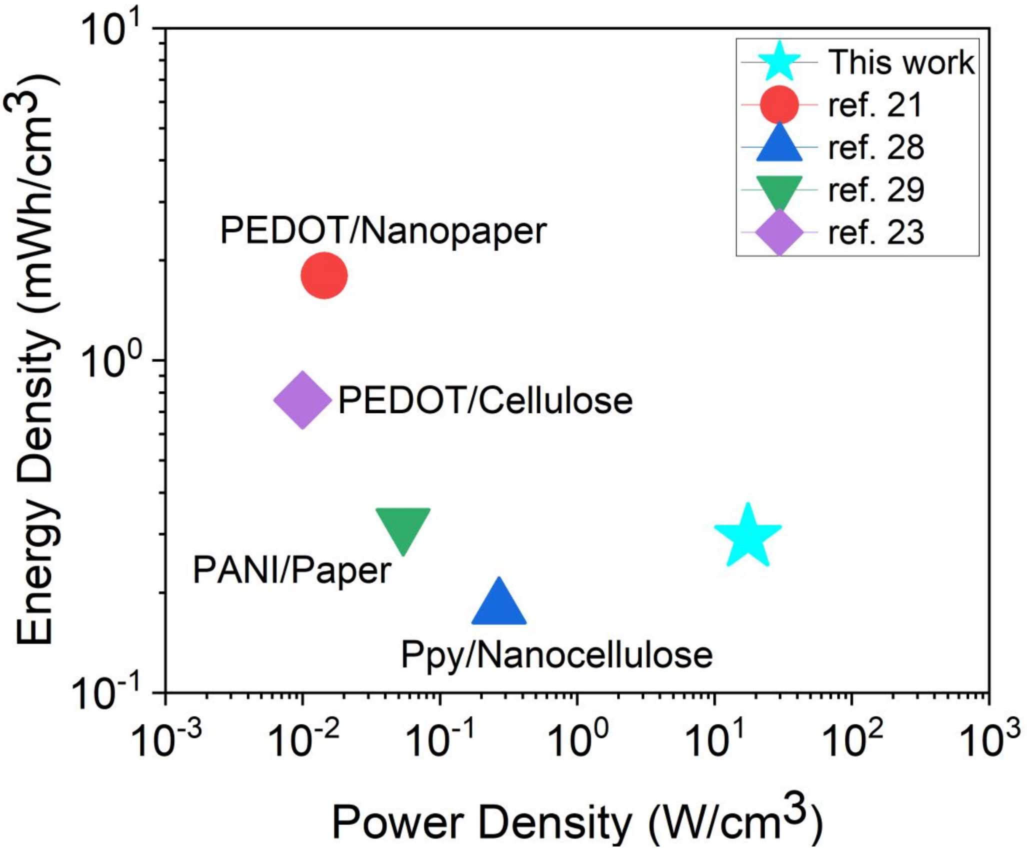

Figure S19. Ragone plot for comparison of paper-based supercapacitor devices [21,23,28,29]

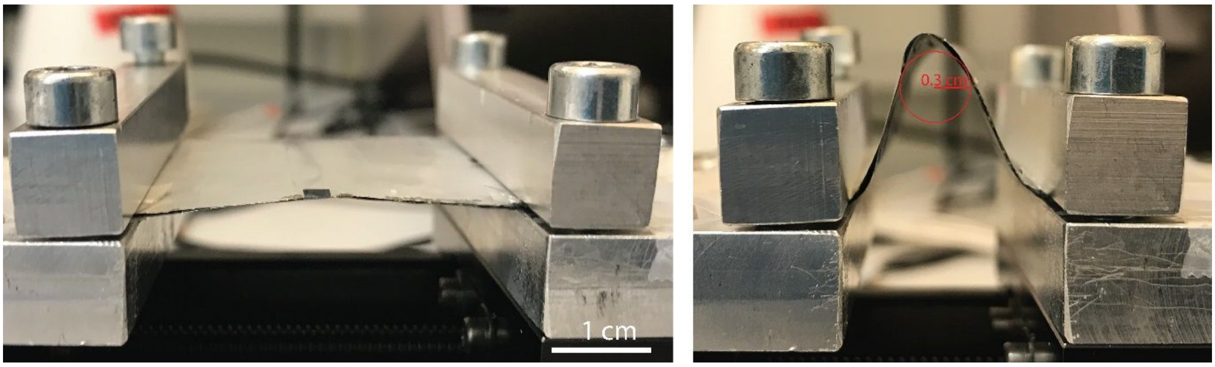

Figure S20. Bending test of paper supercapacitor. left) Flat state, right) Bent state with bending radius (R) of 0.6 cm.

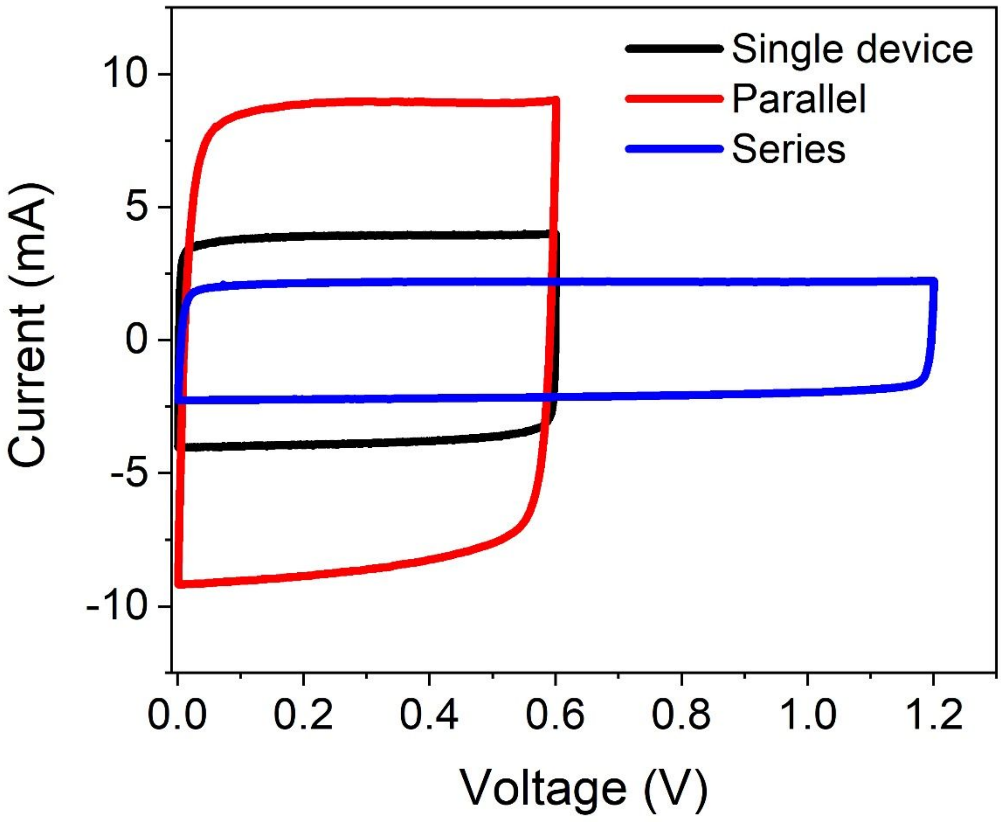

Figure S21. CV (100 mV/s) curves of series and parallel connected two supercapacitors.

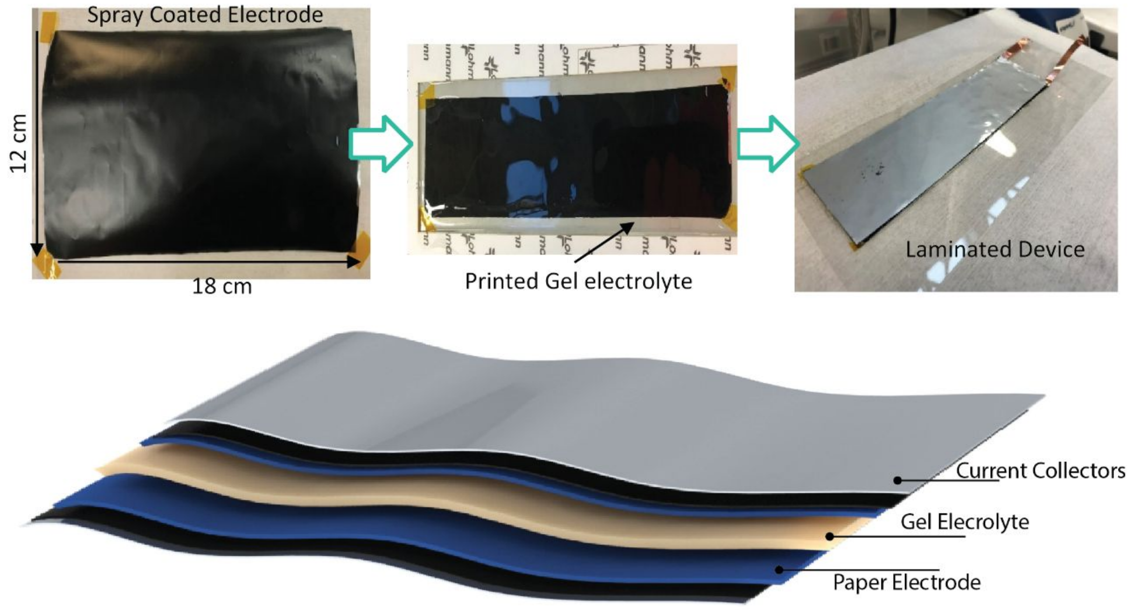

Figure S22. Fabrication Schematics for Large area wearable supercapacitor.

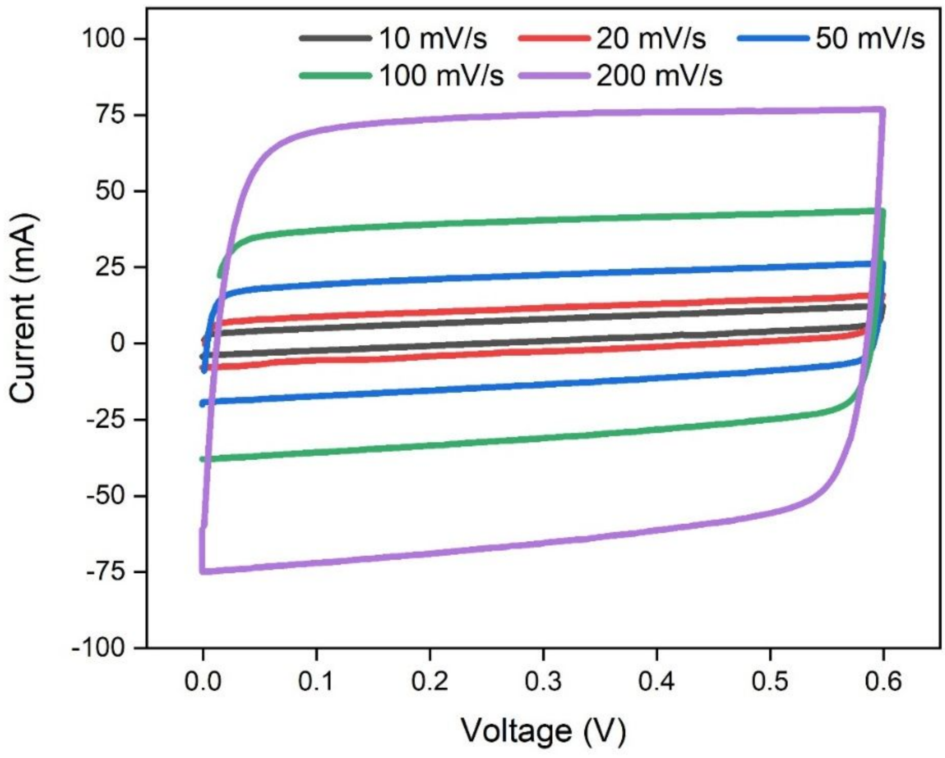

Figure S23. CV of large area, wearable supercapacitor at different scan rates (10-200 mV/s).

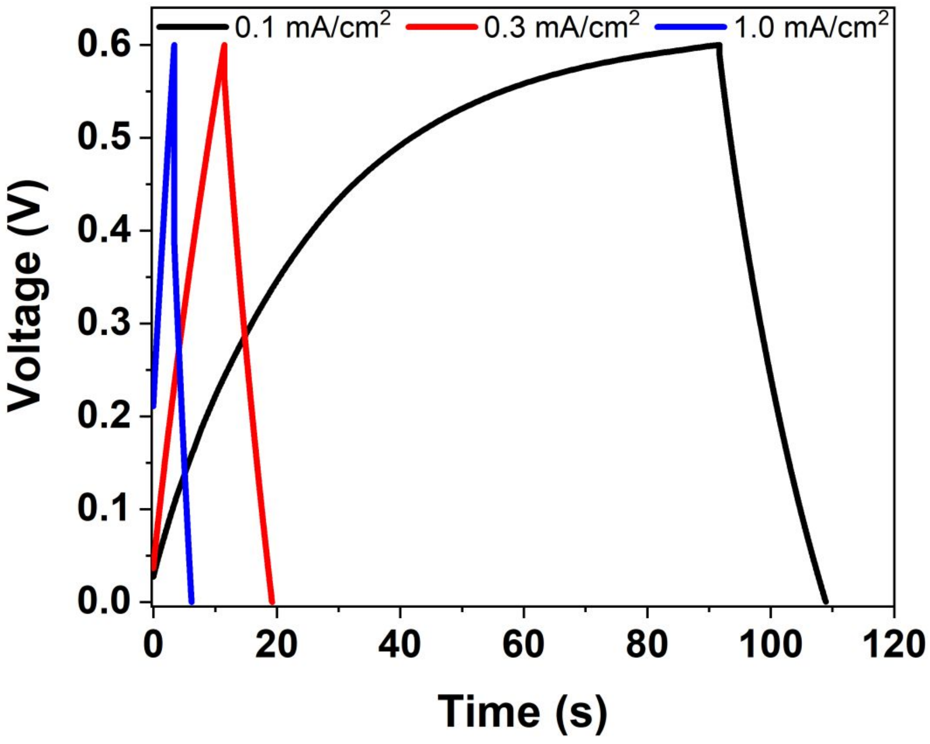

Figure S24. GCD of large area, wearable supercapacitor at different current densities.

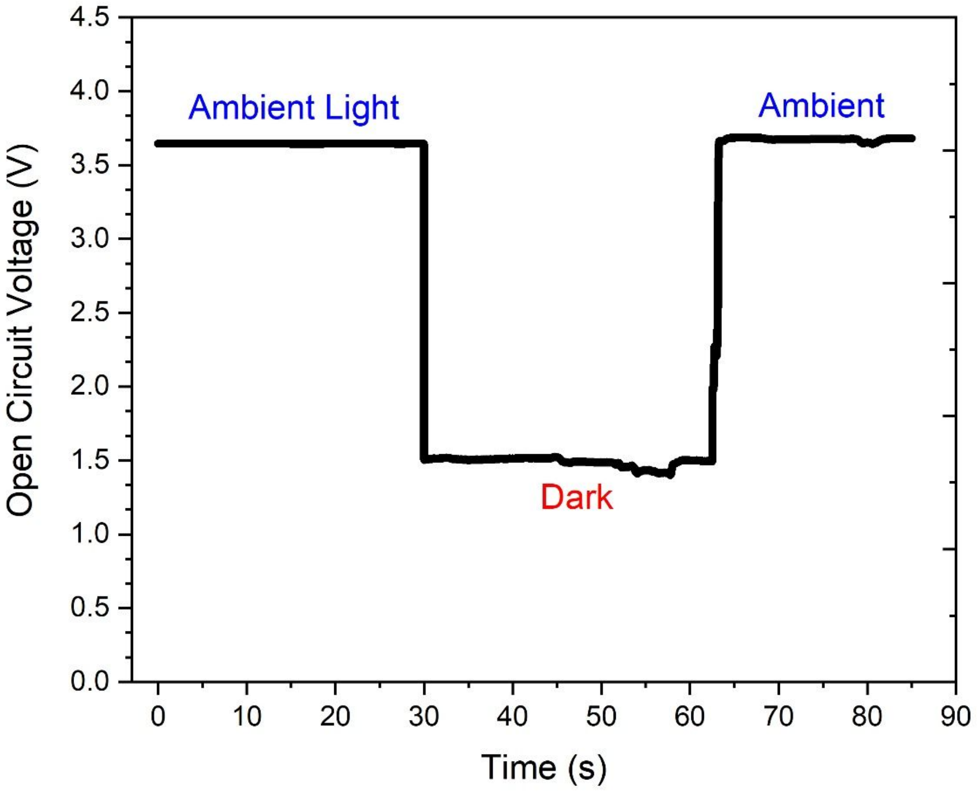

Figure S25. Solar Cell open circuit voltage under different light conditions (Dark room vs.~ 0.1 mW/cm^2).

References

(1) Hammouda, B. A New Guinier-Porod Model. *J Appl Crystallogr* **2010**, *43* (4), 716–719. https://doi.org/10.1107/S0021889810015773.

(2) Anothumakkool, B.; Soni, R.; Bhange, S. N.; Kurungot, S. Novel Scalable Synthesis of Highly Conducting and Robust PEDOT Paper for a High Performance Flexible Solid Supercapacitor. *Energy Environ Sci* **2015**, *8* (4), 1339–1347. https://doi.org/10.1039/c5ee00142k.

(3) Kurra, N.; Park, J.; Alshareef, H. N. A Conducting Polymer Nucleation Scheme for Efficient Solid-State Supercapacitors on Paper. *J. Mater. Chem. A* **2014**, *2* (40), 17058–17065. https://doi.org/10.1039/C4TA03603D.

(4) Diao, Y.; Lu, Y.; Yang, H.; Wang, H.; Chen, H.; D'Arcy, J. M. Direct Conversion of Fe_2O_3 to 3D Nanofibrillar PEDOT Microsupercapacitors. *Adv Funct Mater* **2020**, *30* (32), 2003394. https://doi.org/10.1002/adfm.202003394.

(5) Zhang, J.; Kong, N.; Uzun, S.; Levitt, A.; Seyedin, S.; Lynch, P. A.; Qin, S.; Han, M.; Yang, W.; Liu, J.; Wang, X.; Gogotsi, Y.; Razal, J. M. Scalable Manufacturing of Free-Standing, Strong Ti3C2Tx MXene Films with Outstanding Conductivity. *Advanced Materials* **2020**, *32* (23). https://doi.org/10.1002/adma.202001093.

(6) Wang, X.; Lu, Q.; Chen, C.; Han, M.; Wang, Q.; Li, H.; Niu, Z.; Chen, J. A Consecutive Spray Printing Strategy to Construct and Integrate Diverse Supercapacitors on Various Substrates. *ACS Appl Mater Interfaces* **2017**, *9* (34), 28612–28619. https://doi.org/10.1021/acsami.7b08833.

(7) Wu, Z.-S.; Liu, Z.; Parvez, K.; Feng, X.; Müllen, K. Ultrathin Printable Graphene Supercapacitors with AC Line-Filtering Performance. *Advanced Materials* **2015**, *27* (24), 3669–3675. https://doi.org/10.1002/adma.201501208.

(8) Li, Y.; Ren, G.; Zhang, Z.; Teng, C.; Wu, Y.; Lu, X.; Zhu, Y.; Jiang, L. A Strong and Highly Flexible Aramid Nanofibers/PEDOT:PSS Film for All-Solid-State Supercapacitors with Superior Cycling Stability. *J Mater Chem A Mater* **2016**, *4* (44), 17324–17332. https://doi.org/10.1039/c6ta06981a.

(9) Sumboja, A.; Foo, C. Y.; Wang, X.; Lee, P. S. Large Areal Mass, Flexible and Free-Standing Reduced Graphene Oxide/Manganese Dioxide Paper for Asymmetric Supercapacitor Device. *Advanced Materials* **2013**, *25* (20), 2809–2815. https://doi.org/10.1002/adma.201205064.

(10) Compton, O. C.; Dikin, D. A.; Putz, K. W.; Brinson, L. C.; Nguyen, S. T. Electrically Conductive "Alkylated" Graphene Paper via Chemical Reduction of Amine-Functionalized Graphene Oxide Paper. *Advanced Materials* **2010**, *22* (8), 892–896. https://doi.org/10.1002/adma.200902069.

(11) Wakabayashi, T.; Katsunuma, M.; Kudo, K.; Okuzaki, H. PH-Tunable High-Performance PEDOT:PSS Aluminum Solid Electrolytic Capacitors. *ACS Appl Energy Mater* **2018**, *1* (5), 2157–2163. https://doi.org/10.1021/acsaem.8b00210.

(12) Cheng, T.; Zhang, Y. Z.; Yi, J. P.; Yang, L.; Zhang, J. D.; Lai, W. Y.; Huang, W. Inkjet-Printed Flexible, Transparent and Aesthetic Energy Storage Devices Based on PEDOT:PSS/Ag Grid Electrodes. *J Mater Chem A Mater* **2016**, *4* (36), 13754–13763. https://doi.org/10.1039/c6ta05319j.

(13) Yuan, D.; Li, B.; Cheng, J.; Guan, Q.; Wang, Z.; Ni, W.; Li, C.; Liu, H.; Wang, B. Twisted Yarns for Fiber-Shaped Supercapacitors Based on Wetspun PEDOT:PSS Fibers from Aqueous Coagulation. *J Mater Chem A Mater* **2016**, *4* (30), 11616–11624. https://doi.org/10.1039/c6ta04081k.

(14) Singh, S. B.; Kshetri, T.; Singh, T. I.; Kim, N. H.; Lee, J. H. Embedded PEDOT:PSS/AgNFs Network Flexible Transparent Electrode for Solid-State Supercapacitor. *Chemical Engineering Journal* **2019**, *359* (November 2018), 197–207. https://doi.org/10.1016/j.cej.2018.11.160.

(15) Yu, X.; Su, X.; Yan, K.; Hu, H.; Peng, M.; Cai, X.; Zou, D. Stretchable, Conductive, and Stable PEDOT-Modified Textiles through a Novel In Situ Polymerization Process for Stretchable Supercapacitors. *Adv Mater Technol* **2016**, *1* (2), 1–8. https://doi.org/10.1002/admt.201600009.

(16) Cheng, T.; Zhang, Y.-Z.; Zhang, J.-D.; Lai, W.-Y.; Huang, W. High-Performance Free-Standing PEDOT:PSS Electrodes for Flexible and Transparent All-Solid-State Supercapacitors. *J Mater Chem A Mater* **2016**, *4* (27), 10493–10499. https://doi.org/10.1039/C6TA03537J.

(17) Li, Y.; Ren, G.; Zhang, Z.; Teng, C.; Wu, Y.; Lu, X.; Zhu, Y.; Jiang, L. A Strong and Highly Flexible Aramid Nanofibers/PEDOT:PSS Film for All-Solid-State Supercapacitors with Superior Cycling Stability. *J Mater Chem A Mater* **2016**, *4* (44), 17324–17332. https://doi.org/10.1039/c6ta06981a.

(18) Zhang, M.; Zhou, Q.; Chen, J.; Yu, X.; Huang, L.; Li, Y.; Li, C.; Shi, G. An Ultrahigh-Rate Electrochemical Capacitor Based on Solution-Processed Highly Conductive PEDOT:PSS Films for AC Line-Filtering. *Energy Environ Sci* **2016**, *9* (6), 2005–2010. https://doi.org/10.1039/c6ee00615a.

(19) Li, Z.; Ma, G.; Ge, R.; Qin, F.; Dong, X.; Meng, W.; Liu, T.; Tong, J.; Jiang, F.; Zhou, Y.; Li, K.; Min, X.; Huo, K.; Zhou, Y. Free-Standing Conducting Polymer Films for High-Performance Energy Devices. *Angewandte Chemie - International Edition* **2016**, *55* (3), 979–982. https://doi.org/10.1002/anie.201509033.

(20) Manjakkal, L.; Pullanchiyodan, A.; Yogeswaran, N.; Hosseini, E. S.; Dahiya, R. A Wearable Supercapacitor Based on Conductive PEDOT:PSS-Coated Cloth and a Sweat Electrolyte. *Advanced Materials* **2020**, *1907254*. https://doi.org/10.1002/adma.201907254.

(21) Wang, Z.; Tammela, P.; Huo, J.; Zhang, P.; Strømme, M.; Nyholm, L. Solution-Processed Poly(3,4-Ethylenedioxythiophene) Nanocomposite Paper Electrodes for High-Capacitance Flexible Supercapacitors. *J Mater Chem A Mater* **2016**, *4* (5), 1714–1722. https://doi.org/10.1039/C5TA10122K.

(22) Brooke, R.; Edberg, J.; Say, M. G.; Sawatdee, A.; Grimoldi, A.; Åhlin, J.; Gustafsson, G.; Berggren, M.; Engquist, I. Supercapacitors on Demand: All-Printed Energy Storage Devices with Adaptable Design. *Flexible and Printed Electronics* **2019**, *4* (1). https://doi.org/10.1088/2058-8585/aafc4f.

(23) Li, B.; Lopez-Beltran, H.; Siu, C.; Skorenko, K. H.; Zhou, H.; Bernier, W. E.; Whittingham, M. S.; Jones, W. E. Vaper Phase Polymerized PEDOT/Cellulose Paper Composite for Flexible Solid-State Supercapacitor. *ACS Appl Energy Mater* **2020**, *3* (2), 1559–1568. https://doi.org/10.1021/acsaem.9b02044.

(24) Wang, Z.; Tammela, P.; Huo, J.; Zhang, P.; Strømme, M.; Nyholm, L. Solution-Processed Poly(3,4-Ethylenedioxythiophene) Nanocomposite Paper Electrodes for High-Capacitance Flexible Supercapacitors. *J Mater Chem A Mater* **2016**, *4* (5), 1714–1722. https://doi.org/10.1039/c5ta10122k.

(25) Zhao, D.; Zhang, Q.; Chen, W.; Yi, X.; Liu, S.; Wang, Q.; Liu, Y.; Li, J.; Li, X.; Yu, H. Highly Flexible and Conductive Cellulose-Mediated PEDOT:PSS/MWCNT Composite Films for Supercapacitor Electrodes. *ACS Appl Mater Interfaces* **2017**, *9* (15), 13213–13222. https://doi.org/10.1021/acsami.7b01852.

(26) Liu, R.; Ma, L.; Huang, S.; Mei, J.; Xu, J.; Yuan, G. Large Areal Mass, Flexible and Freestanding Polyaniline/Bacterial Cellulose/Graphene Film for High-Performance Supercapacitors. *RSC Adv* **2016**, *6* (109), 107426–107432. https://doi.org/10.1039/C6RA21920A.

(27) Chen, J.; Chen, H.; Chen, M.; Zhou, W.; Tian, Q.; Wong, C. P. Nacre-Inspired Surface-Engineered MXene/Nanocellulose Composite Film for High-Performance Supercapacitors and Zinc-Ion Capacitors. *Chemical Engineering Journal* **2022**, *428*. https://doi.org/10.1016/j.cej.2021.131380.

(28) Razaq, A.; Nyholm, L.; Sjödin, M.; Strømme, M.; Mihranyan, A. Paper-Based Energy-Storage Devices Comprising Carbon Fiber-Reinforced Polypyrrole-Cladophora Nanocellulose Composite Electrodes. *Adv Energy Mater* **2012**, *2* (4), 445–454. https://doi.org/10.1002/aenm.201100713.

(29) Yao, B.; Yuan, L.; Xiao, X.; Zhang, J.; Qi, Y.; Zhou, J.; Zhou, J.; Hu, B.; Chen, W. Paper-Based Solid-State Supercapacitors with Pencil-Drawing Graphite/Polyaniline Networks Hybrid Electrodes. *Nano Energy* **2013**, *2* (6), 1071–1078. https://doi.org/10.1016/j.nanoen.2013.09.002.
